# PTBP1 is upregulated in response to Zika virus infection and restrains viral replication by hijacking viral NS1 protein to induce NS1 degradation

**DOI:** 10.1128/jvi.01495-25

**Published:** 2026-04-27

**Authors:** Menglan Rao, Yue Kong, Shuang Liu, Zicong Chen, Jiuxiu Lin, Yicong Liang, Zhiwei Lei, Zhen Luo

**Affiliations:** 1 Institute of Medical Microbiology, Department of Immunology and Microbiology, College of Life Science and Technology, Jinan University 47885, Guangzhou, China; 2 Department of Microbiology and Immunology, Basic Medicine College, Jinan University 47885, Guangzhou, China; 3 Department of Dermatology, Second Hospital Affiliated to Guangzhou Medical University, Guangzhou, China; 4 Department of Gastroenterology, Affiliated Qingyuan Hospital, Guangzhou Medical University, Qingyuan People’s Hospital, Qingyuan, China; 5 Key Laboratory of Viral Pathogenesis & Infection Prevention and Control (Jinan University), Ministry of Education, Guangzhou, China; The Ohio State University, Columbus, Ohio, USA

**Keywords:** Zika virus, polypyrimidine tract-binding protein 1, non-structural protein 1, hypoxia-inducible factor-1α, lysosomal pathway

## Abstract

Zika virus (ZIKV), belonging to the *Flaviviridae* family, has been a severe threat to human health since the worldwide outbreak. ZIKV is capable of inducing fetal microcephaly, Guillain-Barré syndrome, and other serious neurological complications. Polypyrimidine tract-binding protein 1 (PTBP1) is a key member of the heterogeneous nuclear ribonucleoproteins (hnRNPs) family, functioning in selective mRNA splicing and gene expression regulation. Our previous study has indicated that the expression of PTBP1 increases in astrocytes upon ZIKV infection, yet the precise regulatory mechanisms underlying its role in viral replication remain elusive. In this study, we elucidated the specific pathway by which ZIKV upregulated PTBP1 expression through the activation of hypoxia-inducible factor-1α (HIF-1α) signal. Further investigation revealed that overexpression of PTBP1 effectively inhibited viral replication, whereas knockdown of PTBP1 significantly enhanced viral replication. Mechanistically, using co-immunoprecipitation assays for protein interaction screening, we identified an interaction between PTBP1 and ZIKV non-structural protein NS1. Detailed studies demonstrated that PTBP1 bound and colocalized with NS1 to lead to the degradation of NS1 protein via a lysosomal pathway. Collectively, our findings unveil a novel mechanism underlying that ZIKV infection induces the expression of PTBP1 via the HIF-1α pathway. Subsequently, the accumulated PTBP1 binds to the ZIKV NS1 protein, promoting NS1 degradation and thereby effectively inhibiting viral replication. The study illustrates a distinct restricted cellular factor that regulates ZIKV replication, which provides a potential target for the control of viral replication and pathogenesis during the ZIKV epidemic.

**IMPORTANCE:**

Since the outbreak of ZIKV infection among humans in 2014, a Zika epidemic has caused Zika fever accompanied by fetal microcephaly, Guillain-Barré syndrome, and other neurological symptoms. Emerging evidence reveals that ZIKV infects astrocytes to specifically induce the expression of polypyrimidine tract-binding protein 1 (PTBP1), one of the hnRNP members. However, the interplay between PTBP1 and ZIKV replication is unclear. Here, we uncover a distinct manner that ZIKV induces PTBP1 expression through the activation of hypoxia-inducible factor-1α (HIF-1α) signal. Additionally, activation of the HIF-1α signal hinders ZIKV replication, relying on PTBP1 accumulation. Further investigations suggest that PTBP1 restrains ZIKV replication by interacting with ZIKV NS1 protein, thereby leading to the degradation of NS1 protein via a lysosomal pathway. Collectively, our findings illustrate a novel restricted cellular factor PTBP1 mediated by HIF-1α that regulates ZIKV replication, which provides a potential therapeutic target of viral replication and pathogenesis against the ZIKV pandemic.

## INTRODUCTION

ZIKV is an arthropod-borne single-stranded positive-sense RNA virus belonging to the Flavivirus genus within the *Flaviviridae* family (1). Although most individuals infected with ZIKV do not exhibit overt symptoms, approximately 20-25% of infected patients typically present with a series of mild clinical manifestations such as headache, fever, rash, and musculoskeletal pain (2). However, the severity of ZIKV infection lies in its potential to cause neurological complications, including fetal microcephaly and neurological defects (3), Guillain-Barré syndrome (4), meningitis, and myelitis (5). Currently, there are no specific vaccines or antiviral drugs available for the prevention or treatment of ZIKV infection, and supportive and symptomatic treatments are the mainstay for managing ZIKV-infected patients (6, 7). It is urgent to discover the exact mechanism that regulates ZIKV replication in the host.

The ZIKV particle is spherical with a diameter of 40–60 nm and a genome size of approximately 10.8 kb, which comprises 5′ and 3′ untranslated regions (UTRs) and a single open reading frame (ORF) (8). The ORF encodes a polyprotein that is cleaved into three structural proteins: C, prM, E, and seven non-structural proteins (NS1, NS2A, NS2B, NS3, NS4A, NS4B, and NS5) (9). Among them, the non-structural proteins play pivotal roles in virus replication, assembly, and evasion of host defenses. The NS1 protein is multifunctional and involved in virus replication and immune evasion (10). It exists in cells as monomers, dimers on the endoplasmic reticulum membrane, and hexamers outside the cell (11). The dimers participate in the formation of virus replication complexes, while the hexamers are involved in immune evasion. ZIKV NS1 and NS4B interact with TANK-binding kinase 1 (TBK1) and inhibit its phosphorylation, thereby suppressing the production of interferon-β (IFN-β) (12). ZIKV NS1 also recruits the host deubiquitinase ubiquitin-specific protease 8 (USP8) to inhibit the degradation of caspase-1, enhancing NOD-, LRR- and pyrin domain-containing protein 3 (NLRP3) inflammasome activation and reducing type I IFN production, thus helping ZIKV evade the host antiviral response (13). Additionally, NS1 serves as an important biomarker for early diagnosis of ZIKV infection (14). Thus, the role of ZIKV NS1 in the control of viral replication is highly concerning.

Polypyrimidine tract-binding protein 1 (PTBP1), also known as PTB or heterogeneous nuclear ribonucleoprotein I (hnRNP I), is a member of the hnRNP family of splicing proteins. With an apparent molecular weight of 57 kDa, it is a shuttling protein primarily located in the nucleus (15). The protein structure consists of four non-canonical RNA recognition motifs, a nuclear localization domain, and a nuclear export signal at the N-terminus (16, 17). PTBP1 participates in RNA maturation, transport, localization, and translation processes, primarily serving as a splicing factor in the alternative splicing of pre-mRNA (18–20). Due to its functional diversity, it has become one of the important targets for studying gene expression regulation mechanisms and disease pathogenesis. PTBP1 is also associated with biological features under infectious conditions. Under hypoxic conditions, HIF-1α can bind to the promoter of mouse PTBP1 to promote its expression, implying that HIF-1α signal plays a regulatory role in PTBP1 expression. Our previous study suggests that the upregulation of PTBP1 accompanies ZIKV infection in primary mouse astrocytes in the host transcriptome analysis (21), but the potential regulation of PTBP1 on ZIKV replication remains elusive.

In this study, we explored the precise mechanism of PTBP1 in the regulation of ZIKV replication. We found that ZIKV infection activated the HIF-1α pathway to induce PTBP1 expression. In turn, the accumulation of PTBP1 bound to ZIKV NS1 protein promoted NS1 degradation, resulting in inhibiting viral replication. The study illustrates that PTBP1 regulates ZIKV replication as a distinct restricted cellular factor in the host, which provides a potential target for interventions in ZIKV replication and pathogenesis.

## MATERIALS AND METHODS

### Cell lines and virus

Mosquito (C6/36) cells, African green monkey kidney (Vero) cells, human lung adenocarcinoma (A549) cells, and human embryonic kidney 293T (HEK293T) cells were purchased from the American Type Culture Collection (ATCC) (Manassas, VA, USA). The cell culture was prepared in Dulbecco’s modified Eagle’s medium (DMEM) (Invitrogen; Carlsbad, CA, USA), 10% fetal bovine serum (FBS) (Gibco; Grand Island, NY, USA), 100 U/mL penicillin, and 100 mg/mL streptomycin (Gibco). All cells were cultured in a humidified incubator at 37°C and 5% CO_2_. ZIKV has previously been described as an Asian strain isolate zl6006 (GenBank accession number KU955589.1) (22), obtained from the Institute of Pathogenic Microorganisms of the Guangdong Provincial Center for Disease Control and Prevention. ZIKV was propagated in C6/36 cells, and the viral titer was measured in Vero cells.

### ZIKV propagation and titration

C6/36 cells were infected with ZIKV stock solution to allow the virus to incubate the cells for 2 h, and then were replaced with fresh DMEM for 6–7 days until the cells caused an obvious cytopathic effect. The cell supernatants were collected and centrifuged at 1,000 × *g* for 3 min. The supernatant was filtered using a 0.22 μm filter to obtain the virus stock solution. The TCID_50_ assay was used to calculate the ZIKV titer. Vero cells were incubated with ZIKV at an appropriate multiplicity of infection (MOI) for 2 h. The culture medium was replaced with fresh medium to continue culture for 3–5 days. After reaching the predetermined time point, the cells were observed for subsequent analysis. The effect of viral infection on the control group was compared and evaluated. The Reed Muench method was used to calculate the plaque-forming unit (PFU) of the virus.

### Antibodies, reagents, and plasmids

Mouse monoclonal antibody against GAPDH (Cat #G9295) was purchased from Gibco. Rabbit polyclonal antibodies against PTBP1 (Cat #12582-1-AP), Vinculin (Cat #12582-1-AP 26520-1-AP) and HIF-1α (Cat #20960-1-AP), rabbit IgG control polyclonal antibody (Cat #30000-0-AP), rabbit monoclonal antibody against HA (Cat #51064-2-AP), Histone H3 (Cat #68345-1-Ig), HRP-conjugated Affinipure goat anti-mouse IgG (Cat #SA00001-1), and goat anti-mouse IgG (Cat #SA00001-2) were obtained from Proteintech Group (Chicago, IL, USA). Rabbit polyclonal antibody against phospho-HIF-1α (Ser641/Ser643) (Cat #TA0062) was obtained from Abmart (Shanghai, China). Rabbit polyclonal antibodies against ZIKV NS5 (Cat #GTX133312) and NS1 (Cat #GTX634159) were obtained from Genetex Inc. (Irvine, CA, USA). Mouse monoclonal antibody against FLAG (Cat #F3165) was purchased from Sigma (St Louis, MO, USA). Rabbit monoclonal antibodies against TBK1 (Cat #3504), phospho-TBK1 (Ser172) (Cat #5483), and LAMP1 (Cat #9091) were purchased from Cell Signaling Technology (Danvers, MA, USA). FITC-conjugated Affinipure donkey anti-rabbit IgG (Cat #SA00003-8) and CoraLite594-conjugated donkey anti-mouse IgG (Cat #SA00013-7) were purchased from Proteintech. IOX2 (Cat #HY-15468), YC-1 (Cat #HY-14927), 3-MA (Cat #HY-19312), MG132 (Cat #HY-13259), and NH_4_Cl (Cat #HY-Y1269C) were purchased from MedChemExpress (Princeton, NJ, USA). LysoTracker Deep Red (Cat #L12492) was purchased from Invitrogen (Carlsbad, CA, USA). DAPI (Cat #10236276001) was purchased from Roche (Basel, Switzerland).

The cDNA encoding human PTBP1, NLS deletion (15–41 aa), and Q154A mutants cloned into pCAGGS-HA vector was constructed by a standard molecular cloning method. Human HIF-1α gene was ligated to pcDNA3.1-Myc-His vector (Thermo Fisher Scientific, Carlsbad, CA, USA). The PTBP1 promoter (−1,200 to +1 bp) was amplified from the genome of A549 cells. The WT and mutant PTBP1 promoters were cloned into pGL3-basic vector (Promega, Madison, WI, USA). The plasmids encoding ZIKV non-structural proteins, FLAG-NS1, NS3, NS4A, NS4B, NS5, or pcDNA3.1 (+)−3 × FLAG vector were previously described (23). The bimolecular fluorescence complementation (BiFc) assay was performed to examine physical protein-protein interaction as previously described (24, 25). The DNA fragment of the ZIKV NS1 gene was ligated into the pBiFc-VC155 vector (plasmid Cat #22011) (Addgene, Cambridge, MA, USA) to express the NS1 fusion protein, while PTBP1 gene was cloned into the pBiFc-VN173 vector (plasmid Cat #22010) (Addgene) to express the PTBP1 fusion protein.

### RNA extraction and qRT-PCR

Cells were lysed using TRIzol reagent (Invitrogen) for 10 min at room temperature, and total RNA was extracted. Then, a certain amount of RNA sample was generated to cDNA using the reverse transcription kit (Cat #RRO36A; Takara Bio Inc). The cDNA samples in triplex were subjected to qPCR detection by Light Cycler 480 (Roche Diagnostics) and SYBR Green Real-Time PCR Master Mix (Roche Di Biologists). The value was calculated by using 2^−△△CT^, and all data were normalized to GAPDH. The primers used in this study are as follows:

GAPDH F: 5′-GTCTCCTCTGACTTCAACAGCG;

GAPDH R: 5′-ACCACCCTGTTGCTGTAGCCAA;

PTBP1 F: 5′-CTCCAAGTTCGGCACAGTGTTG;

PTBP1 R: 5′-CAGGCGTTGTAGATGTTCTGCC;

shPTBP1 F: 5′-CCGGGCCAACACCATGGTGAACTACCTCGAGGTAGTTCACCATGGTGTTGGCTTTTTG;

shPTBP1 R: 5′-AATTCAAAAAGCCAACACCATGGTGAACTACCTCGAGGTAGTTCACCATGGTGTTGGC;

ZIKV F: 5′-GGTCAGCGTCCTCTCTAATAAACG; and

ZIKV R: 5′- GCACCCTAGTGTCCACTTTTTCC.

### Lentiviral packaging

HEK293T cells were transfected with psPAX2, pMD2.G, and lentiviral vector plasmid (pLenti or pLKO.1) containing the target gene or empty plasmid. After 48 h, the lentiviral supernatant was collected and centrifuged at 1,500 × *g* for 5 min. The supernatant was filtered through a 0.45 μm filter. Lentiviral infection solution containing an appropriate amount of polyglutamine was prepared and added to the cells for 48 h of continuous infection. The cells were incubated with 0.5 μg/mL puromycin for 1 week until the cells were no longer dead, and then the expression efficiency was measured by Western blot.

### Western blot

Cells were lysed in RIPA buffer for 1 h at 4°C and then centrifuged at 4°C, 13,000 × *g* for 10 min to collect the cell lysate. The protein concentration was measured using the BCA assay kit (Beyotime Biotechnology, Haimen, Jiangsu, China). Proteins were separated by SDS-PAGE and transferred to the PVDF membrane. The membrane was blocked with TBST containing 5% skimmed milk powder for 1 h at room temperature. After three washes with TBST, the membranes were incubated with primary antibody overnight at 4°C. After the final wash with TBST, the membranes were incubated with secondary antibodies for 1 h. The protein bands were captured using a chemiluminescence imaging system (Bio-Rad, Hercules, CA, USA). The protein expression relative to the internal control is quantified using ImageJ software.

### Reactive oxygen species (ROS) detection

For intracellular ROS measurement, A549 cells were incubated with 10 μM 2′,7′-dichlorodihydrofluorescein diacetate (DCFH-DA) (Cat #S0033S) (Beyotime Biotech Inc., Shanghai, China) according to the manufacturer’s instructions. The fluorescence of the oxidized product 2′,7′-dichlorofluorescein (DCF) was quantified by flow cytometry (Thermo Fisher Scientific).

### Chromatin immunoprecipitation (ChIP)

Cells were fixed in 1% formaldehyde for 10 min at room temperature with gentle rotation, and the reaction was quenched with 0.125 M glycine for 5 min. After two rinses with ice-cold PBS, cells were lysed using the Pierce Magnetic ChIP Kit (Thermo Fisher Scientific) according to the manufacturer’s instructions. Chromatin was fragmented by sonication to ~200–500 bp (optimized to predominantly 200–300 bp). The sheared chromatin was incubated overnight at 4°C with an anti-phospho-HIF-1α (Ser641/Ser643) antibody; normal IgG served as the negative control. Antibody–chromatin complexes were captured with Protein A/G magnetic beads and washed sequentially with low-salt, high-salt, LiCl, and Tris-EDTA (TE) buffers. Bound material was eluted in 1% SDS and 0.1 M NaHCO_3_, and cross-links were reversed by incubation with 200 mM NaCl at 65°C overnight. DNA was purified using the kit’s spin columns or by phenol–chloroform extraction and analyzed by quantitative PCR (qPCR) with primers targeting the regions of *PTBP1* promoter. The primers used were as follows: Region 1 F: 5′-GCCTTGAGGAATAACCGCCT-3′; Region 1 R: 5′-GAAATCGCAGGCGCCTATTG-3′; Region 2 F: 5′-AATAGGCGCCTGCGATTTCT-3′; and Region 2 R: 5′-CACAAAATGGCGGAGACGC-3′.

### Luciferase reporter assay

Luciferase reporter assay was processed by using Firefly Luciferase Reporter Assay System (Cat #E1500) (Promega) according to the manufacturer’s instructions. A549 cells were seeded in 24-well plates and were co-transfected with HIF-1α expressing plasmid and indicated WT and mutant PTBP1 promoter plasmids for 24 h. Harvested cells were lysed, and relative luciferase activities were measured using a Varioskan LUX multimode microplate reader (Thermo Fisher Scientific).

### Co-immunoprecipitation assay

HEK293T cells were transfected with the indicated plasmids and harvested 36 h later. The cells were lysed with RIPA lysis buffer containing protease inhibitors and then centrifuged at 12,000 × *g*, 4°C for 10 min. The cell lysates were incubated with FLAG-Trap Protein-G Sepharose (GE Healthcare, Milwaukee, WI, USA) solution for 4 h. The pellets were washed with lysis buffer. The samples were suspended by adding 2 × loading buffer and subjected to Western blot analysis.

### Immunofluorescence assay

Cells were fixed with 4% paraformaldehyde for 30 min and washed three times with PBS for 5 min each time. Cells were permeabilized with 0.4% Triton X-100 for 20 min and washed three times with PBS for 5 min each time. Then, a 5% BSA blocking solution was added and blocked at room temperature for 30 min. After blocking, cells were incubated with primary antibodies, then fluorescent secondary antibodies and DAPI. For the staining of endogenous PTBP1 and ZIKV NS1, two rabbit antibodies were labeled with green and red dyes, respectively, with a multiplex immunohistochemistry (IHC)/immunofluorescence (IF) staining kit (Cat #RK05903; ABclonal Biotech, Wuhan, China) according to the manufacturer’s instructions. After cells were washed and observed under a confocal laser scanning microscope (Leica TCS SP8; Heidelberg, Germany).

### Nuclear and cytoplasmic fractionation

Nuclear and cytoplasmic proteins were prepared using a Nuclear and Cytoplasmic Protein Extraction Kit (Cat #PC306) (Epizyme, Shanghai, China) in accordance with the manufacturer’s instructions. Fractionated proteins were resolved by SDS–PAGE and analyzed by Western blot. Histone H3 and GAPDH served as nuclear and cytoplasmic markers, respectively.

### Lysosome isolation

Lysosomes were isolated using a lysosomal extraction kit (Cat #EX2670) (Solarbio, Beijing, China). Briefly, cells were washed three times with PBS, incubated with 400 μL ice-cold Reagent A for 10 min at 4°C, homogenized 30–40 strokes with a Dounce homogenizer, and centrifuged at 1,000 × *g* for 5 min at 4°C. The supernatant was then centrifuged sequentially at 3,000 × *g* and 5,000 × *g* for 10 min each; pellets were discarded, and the supernatants were pooled. The resulting supernatant was centrifuged at 25,000 × *g* for 20 min, and the pellet was resuspended in 400 μL ice-cold Reagent B. The suspension was subsequently centrifuged at 25,000 × *g* for 20 min at 4°C, and the lysosomal pellet was resuspended in 100 μL Lysosome Preservation Solution C for downstream experiments. Fractionated proteins were separated by SDS–PAGE and analyzed by Western blot. LAMP1 was used as the lysosomal marker and GAPDH as the cytoplasmic marker.

### Statistical analysis

All data were reproducible to obtain similar results. The statistical significance of the comparison of the two means was evaluated by an unpaired Student’s *t*-test. GraphPad Prism 8 software (San Diego, CA, USA) was used for analysis. The statistical significance is expressed as follows: *, *P* < 0.05; **, *P* < 0.01; and ***, *P* < 0.001.

## RESULTS

### ZIKV infection specifically induces PTBP1 expression in A549 cells

**Fig 1:**
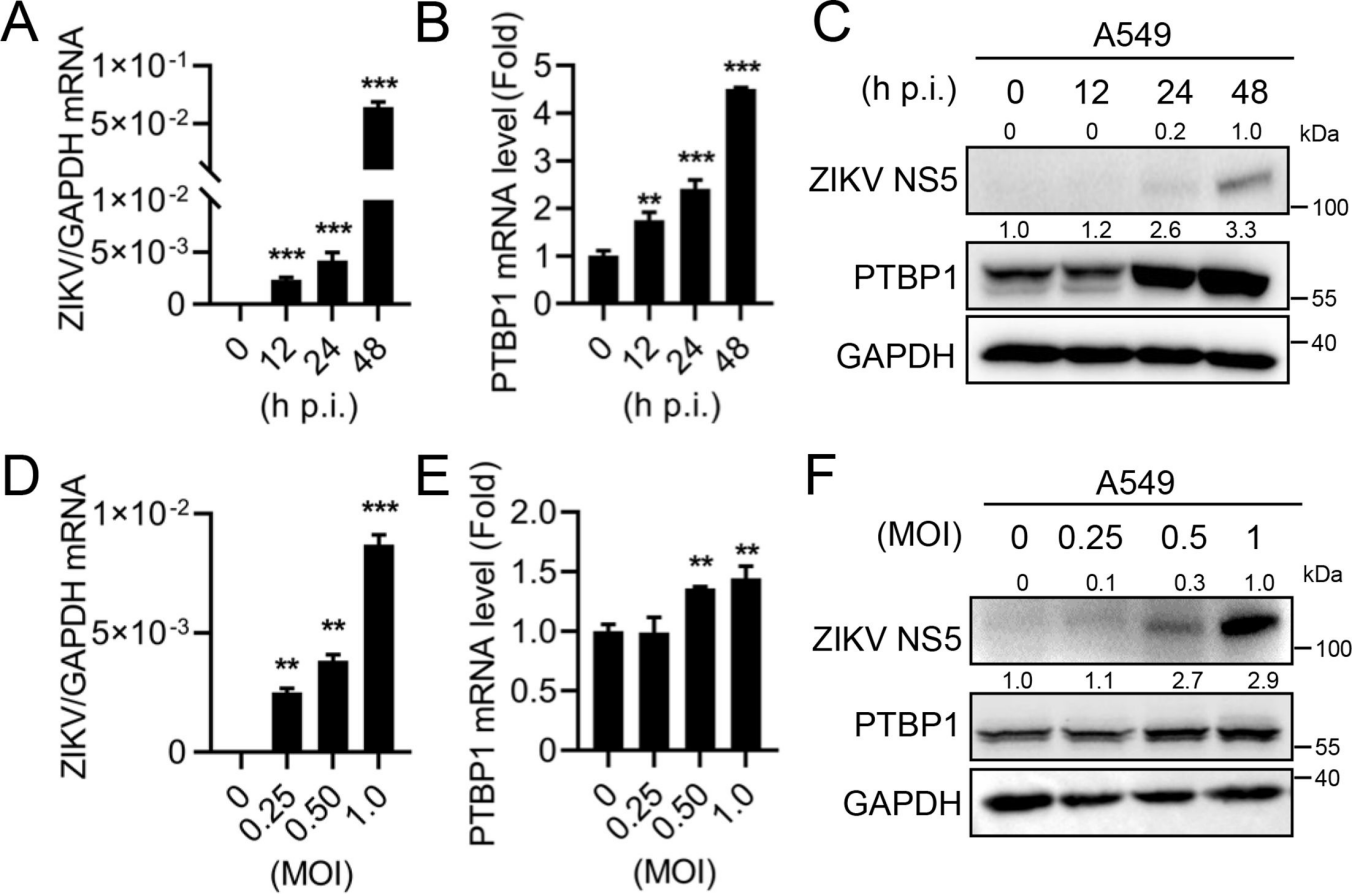
ZIKV infection upregulates PTBP1 expression in A549 cells. (**A–C**) A549 cells were infected with ZIKV at an MOI of 1 for different durations (0, 12, 24, and 48 h). The cells were collected for ZIKV RNA level detected by qRT-PCR (**A**), PTBP1 mRNA (**B**), and protein (**C**) levels detected by qRT-PCR and Western blot, respectively. (**D–F**) A549 cells were infected with ZIKV at MOIs of 0, 0.25, 0.5, and 1.0 for 24 h. ZIKV RNA level was detected by qRT-PCR (**A**), while PTBP1 mRNA (**B**) and protein (**C**) levels were detected by qRT-PCR and Western blot, respectively. Graphs were expressed as Mean ± SD, *n* = 3. ns, not significant; **, *P* < 0.01; ***, *P* < 0.001.

To investigate the effect of ZIKV infection on the expression of PTBP1 in A549 cells, we first infected the cells with ZIKV for varying durations. The results displayed that ZIKV RNA level increased from 12 to 48 h p.i. (Fig. 1A), suggesting a robust viral replication in A549 cells. Meanwhile, the mRNA (Fig. 1B) and protein (Fig. 1C) levels of PTBP1 were unregulated from 12 to 48 h p.i. upon ZIKV infection, indicating that ZIKV induces PTBP1 expression in a time-dependent manner. Consistently, we then infected the cells with ZIKV for varying multiplicities of infection (MOIs). ZIKV RNA level increased at an MOI from 0.25 to 1.0 (Fig. 1D). The mRNA (Fig. 1E) and protein (Fig. 1F) levels of PTBP1 were induced at an MOI from 0.25 to 1.0 upon ZIKV infection, indicating that ZIKV induces PTBP1 expression in a dose-dependent manner. These findings collectively confirm that ZIKV infection typically induces PTBP1 expression in A549 cells.

### ZIKV infection upregulates PTBP1 expression by activating the HIF-1α signal

**Fig 2:**
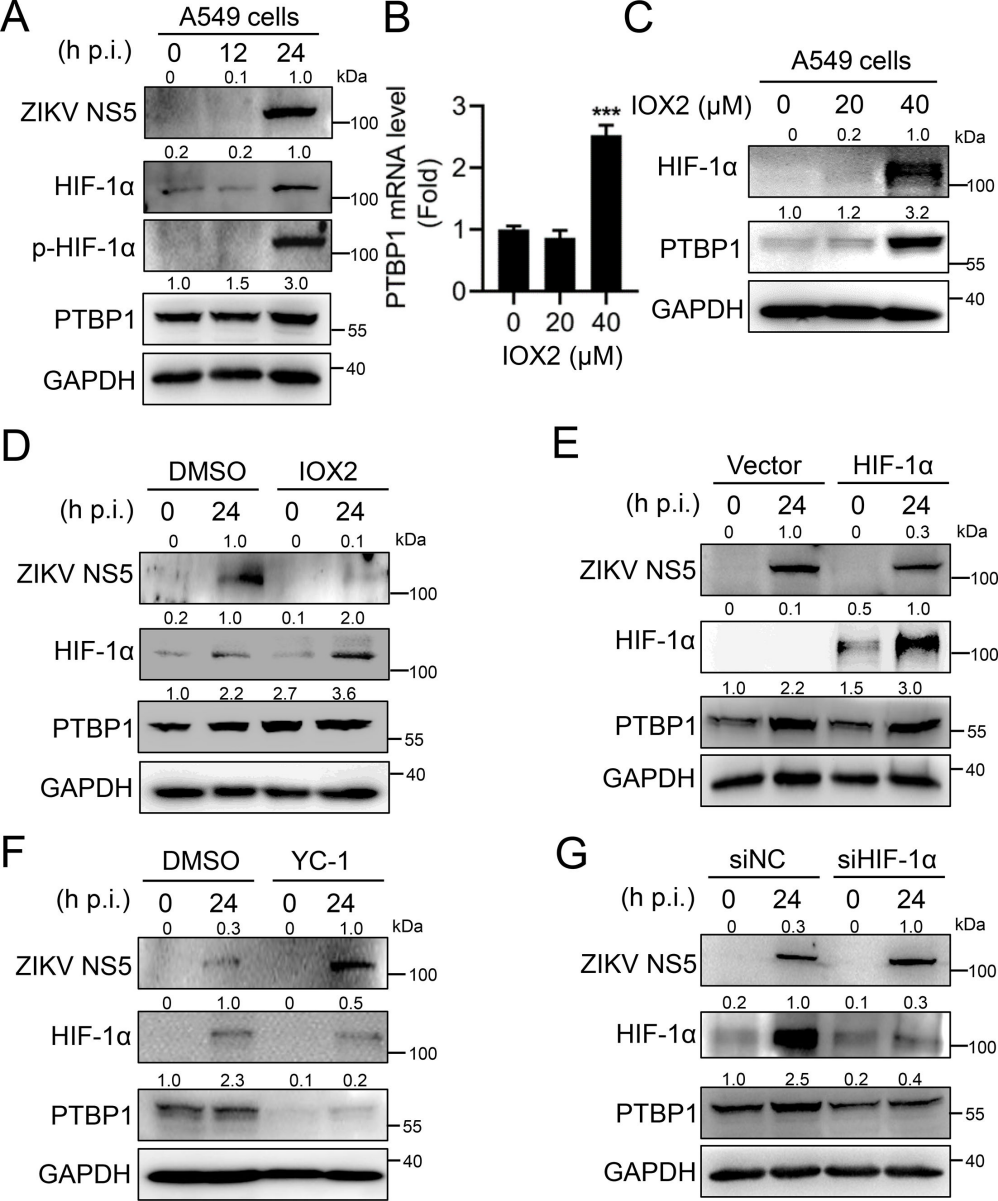
ZIKV infection promotes PTBP1 expression via HIF-1α signal. (**A**) A549 cells were infected with ZIKV at an MOI of 1 at different time points (0, 12, and 24 h). Cells were collected at the corresponding time points for Western blot analysis. (**B and C**) A549 cells were stimulated with different doses of IOX2 (0, 20, and 40 μM) for 12 h. Cells were collected for qRT-PCR (**B**) and Western blot (**C**) analysis, respectively. (**D and E**) A549 cells were transfected with pcDNA-3.1-HIF-1α-Myc-His (2 μg) (**D**) or stimulated with IOX2 (40 μM) (**E**) for 12 h, followed by infection with ZIKV (MOI = 1) for an additional 24 h. Cells were collected for Western blot analysis, respectively. (**F and G**) A549 cells were transfected with siRNA targeting HIF-1α (siHIF-1α) (50 nM) (**F**) or stimulated with a HIF-1α inhibitor YC-1 (20 μM) (**G**) for 16 h, followed by infection with ZIKV (MOI = 1) for another 24 h. Cells were collected for Western blot analysis, respectively. Graphs were expressed as Mean ± SD, *n* = 3. ns, not significant; **, *P* < 0.01; ***, *P* < 0.001.

The specific mechanism by which ZIKV upregulates the expression of PTBP1 remains unclear. Hypoxic and cellular stress responses are triggered by a process that depends on the mediation of HIF-1α (26). Under hypoxic conditions, HIF-1α can bind to the promoter of mouse PTBP1 to promote its expression (27). Based on this, we postulated that ZIKV upregulates PTBP1 by inducing oxidative stress to activate HIF-1α. Upon ZIKV infection in A549 cells, the expressions of both HIF-1α and PTBP1 proteins were upregulated as ZIKV replicated in a time-dependent fashion (Fig. 2A). Notably, ZIKV infection initiated the phosphorylation of HIF-1α (Fig. 2A), implying ZIKV infection activates the HIF-1α signal. To confirm the effect of HIF-1α activation on the PTBP1 expression, A549 cells were stimulated with an HIF-1α modulator IOX2. The expression of both PTBP1 RNA (Fig. 2B) and protein (Fig. 2C) increased with the growing IOX2 concentrations.

To further investigate the role of the HIF-1α signal on the induction of PTBP1 during ZIKV infection, A549 cells were stimulated with IOX2, followed by infection with ZIKV. When HIF-1α is activated by IOX2 before ZIKV infection, HIF-1α signal enhanced ZIKV-induced PTBP1 expression in both RNA (Fig. S1A) and protein (Fig. 2E) levels. Similarly, A549 cells were transfected with HIF-1α plasmid and then infected with ZIKV. HIF-1α overexpression promoted ZIKV-induced PTBP1 expression in both RNA (Fig. S1B) and protein (Fig. 2E) levels. Notably, IOX2-activated HIF-1α and HIF-1α overexpression restrained ZIKV NS5 expression (Fig. 2D and E), suggesting the suppression of viral replication by HIF-1α. In contrast, when the HIF-1α signal was inhibited by an inhibitor YC-1, the upregulation of PTBP1 RNA (Fig. S1C) and protein (Fig. 2F) induced by ZIKV was significantly suppressed. In parallel, knockdown of HIF-1α using siRNA inhibited ZIKV-induced PTBP1 expression in both RNA (Fig. S1D) and protein (Fig. 2G) levels. Importantly, YC-1-inhibited HIF-1α and HIF-1α knockdown promoted ZIKV NS5 expression (Fig. 2F and G), suggesting an inhibitory role of HIF-1α on viral replication. Thus, these results support the evidence that ZIKV upregulates PTBP1 expression by activating the HIF-1α signal.

### HIF-1α transcriptionally upregulates PTBP1 expression

**Fig 3:**
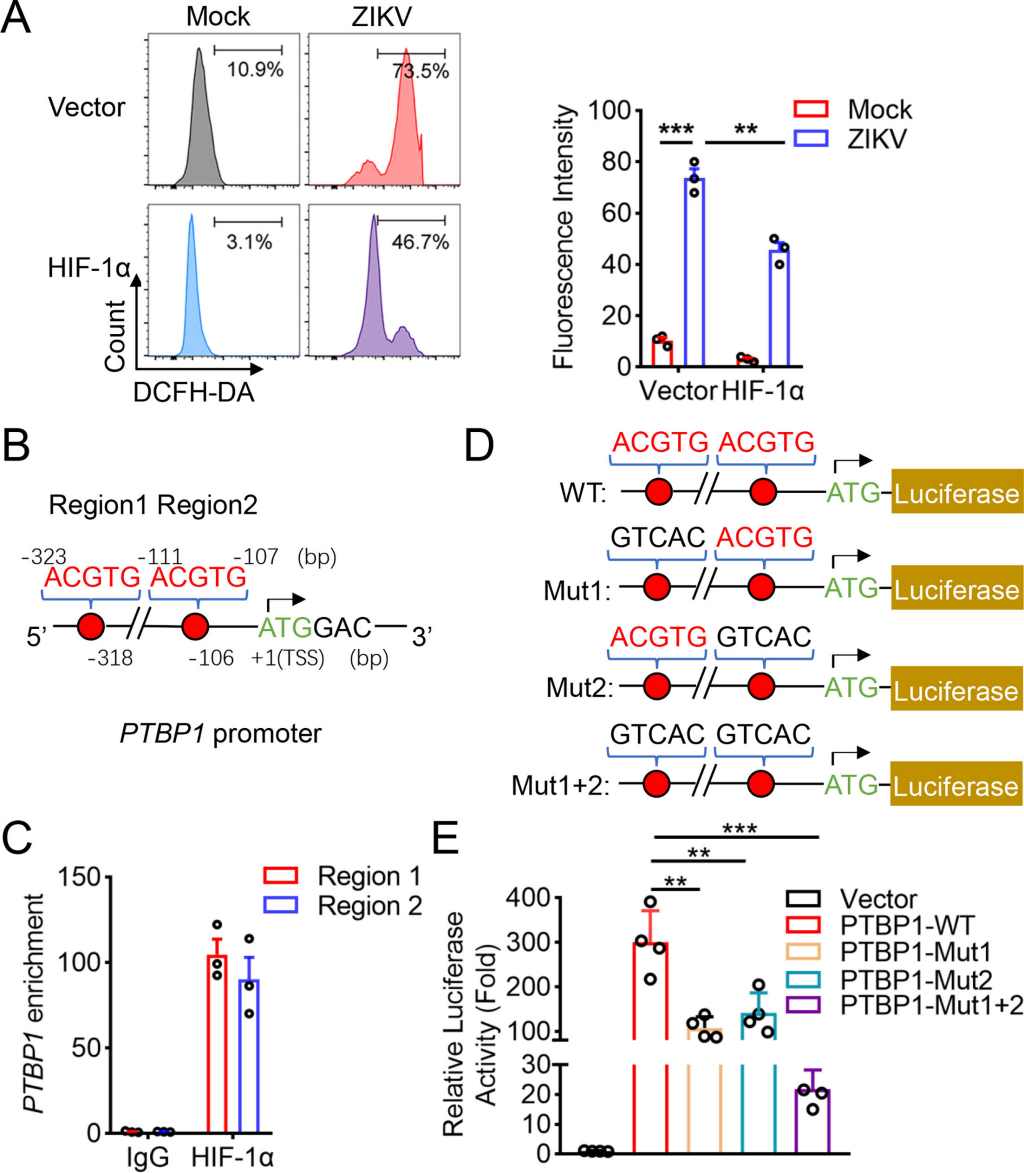
The transcriptional regulation of PTBP1 by HIF-1α. (**A**) A549 cells were transfected with 1.0 μg of each plasmid: Vector or pcDNA-3.1-HIF-1α-Myc-His. At 24 h post-transfection, cells were infected with ZIKV (MOI = 1) for 24 h, and the production of ROS was measured by the MFI of 2′,7′-dichlorofluorescein (DCF) by flow cytometry. (**B**) Schematic diagram of the *PTBP1* promoter with two HIF-1α binding sites. (**C**) Chromatin prepared from A549 cells was immunoprecipitated with control IgG or phospho-HIF-1α antibodies, followed by qPCR with PTBP1 primers flanking HIF-1α responsive genomic sequence using ChIP DNA. (**D**) Schematic diagram of the predicted HIF-1α binding sites in the *PTBP1* promoter. Matches in seed regions are indicated by red letters. The three mutations (black; Mut1, Mut2, and Mut1+2) were introduced into the binding sites of HIF-1α that were subcloned into the pGL3-basic plasmid for the luciferase reporter assay. (**E**) A549 cells were co-transfected with pcDNA-3.1-HIF-1α-Myc-His and pGL3-basic vector or pGL3-PTBP1-promoter (WT and mutants) for 24 h. Cells were lysed, and luciferase activities were measured. Graphs were expressed as Mean ± SD, *n* ≥ 3. ns, not significant; **, *P* < 0.01; ***, *P* < 0.001.

HIF-1α plays a critical role in the mammalian response to reactive oxygen species (ROS) (28). Considering ZIKV infection activates HIF-1α signal, we first explored whether ROS induced ZIKV-driven HIF-1α expression. A549 cells were infected with ZIKV, and ROS measurement in live and fixed cells was performed using fluorescent tools. We found that ROS production was dramatically increased upon ZIKV infection (Fig. 3A). However, in the presence of HIF-1α, ZIKV-induced ROS production dropped (Fig. 3B), suggesting HIF-1α suppressed ZIKV replication and virus-induced ROS production. Then, the regulatory mechanism of PTBP1 expression involved in the HIF-1α signal was investigated. Using an informative resource for detecting transcription factor binding sites (TFBSs) in *PTBP1* promoter, we predicted two HIF-1α binding sites at −323 to −319 (ACGTG) and −111 to −107 (ACGTG) bp of *PTBP1* promoter (Fig. 3B). Usually, phosphorylation of HIF-1α acts as a transcriptional factor. The chromatin immunoprecipitation (ChIP) experiment achieved optimal enrichment and yield of two regions of immunoprecipitated DNA by anti-phospho-HIF-1α antibody (Fig. 3C), indicating a specific binding of transcriptional factor HIF-1α in *PTBP1* promoter. To further estimate the transcriptional upregulation of PTBP1 by HIF-1α, we constructed the luciferase reporter based on the *PTBP1* promoter (Fig. 3D) and then co-expressed with HIF-1α. In wild-type (WT) *PTBP1* promoter reporter, the luciferase activity was increased as expected compared to the control vector (Fig. 3E); however, when in either mutant of site 1, site 2, or both sites with *PTBP1* promoter reporter, the increased luciferase activity was significantly restored (Fig. 3E). Thus, HIF-1α binds to the *PTBP1* promoter to transcriptionally upregulate PTBP1 expression.

### PTBP1 inhibits ZIKV replication

**Fig 4:**
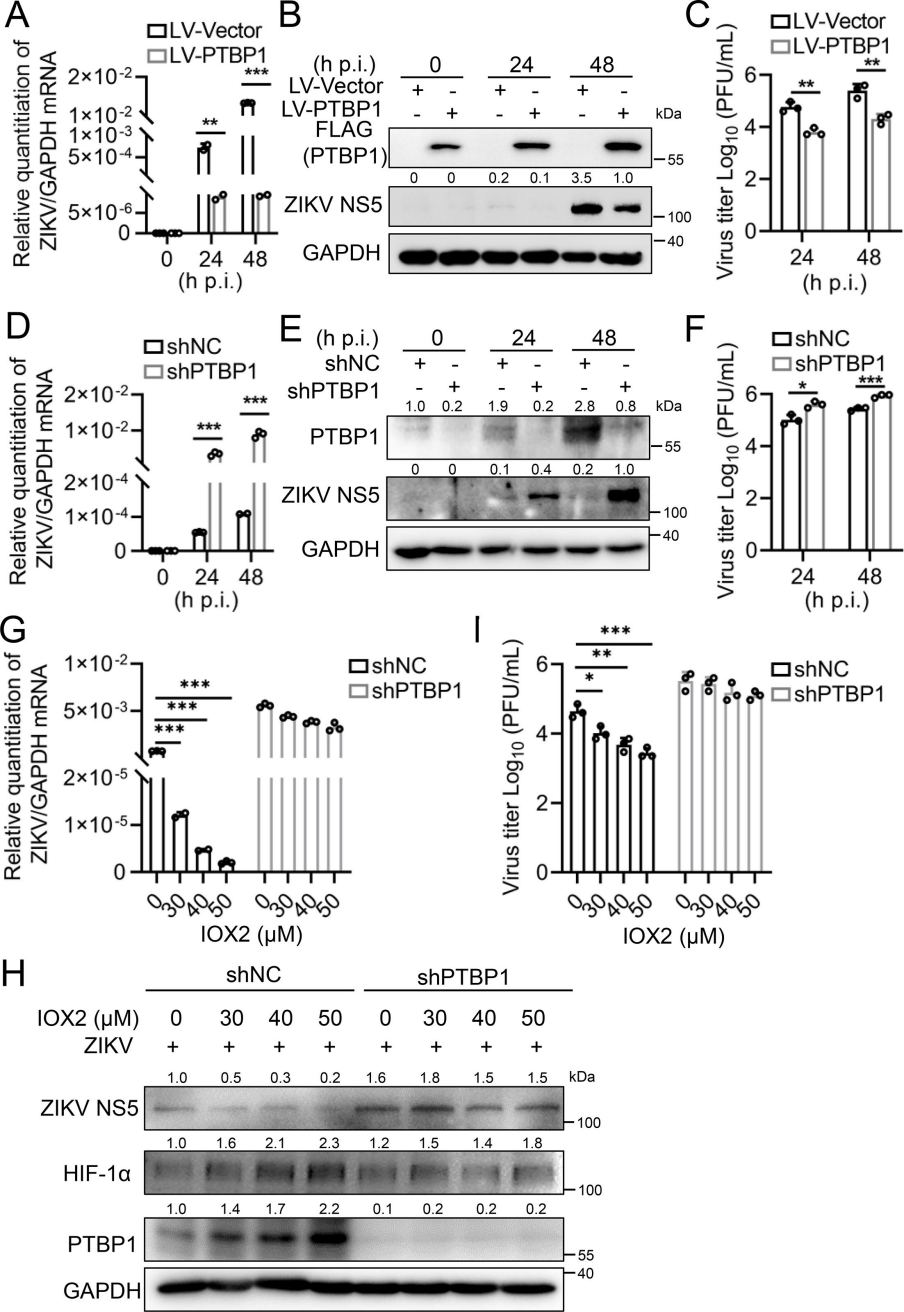
PTBP1 suppressed ZIKV replication. (**A–C**) LV-PTBP1 and LV-Vector cells were infected with ZIKV (MOI = 1) at different time points (0, 24, and 48 h). Cells were collected at the corresponding time points for qRT-PCR (**A**) and Western blot (**B**) analysis, respectively. Supernatants were subjected to TCID_50_, and the virus titer was calculated (**C**). (**D–F**) shPTBP1 and shNC cells were infected with ZIKV (MOI = 1) at different time points (0, 24, and 48 h). Cells were collected at the corresponding time points for qRT-PCR (**D**) and Western blot (**E**) analysis, respectively. Supernatants were subjected to TCID_50_, and the virus titer was calculated (**F**). (**G–I**) shPTBP1 cells and shNC control cells were treated with different concentrations of IOX2 (0, 30, 40, 50 μM) for 12 h and then infected with ZIKV (MOI = 1). Cells were collected 24 h post-infection for qRT-PCR (**G**) and Western blot (**H**) analysis, respectively. Supernatants were subjected to TCID_50_, and the virus titer was calculated (**I**). Graphs were expressed as Mean ± SD, *n* = 3. *, *P* < 0.05; **, *P* < 0.01; and ***, *P* < 0.001.

Since the upregulation of PTBP1 is affected by ZIKV infection, suggesting a pivotal role of PTBP1 during ZIKV replication. To this end, we constructed a FLAG-tagged PTBP1-expressing lentivirus and successfully established an A549 cell line stably expressing PTBP1 (LV-PTBP1), along with a control cell line (LV-Vector). The overexpression of PTBP1 was verified by Western blot analysis (Fig. S2A). In the LV-PTBP1 A549 cells infected with ZIKV, we observed that overexpression of PTBP1 (Fig. S2B) significantly reduced the levels of ZIKV RNA (Fig. 4A) and viral NS5 protein (Fig. 4B) relative to the LV-Vector A549 cells infected with ZIKV. Furthermore, TCID_50_ assays confirmed that overexpression of PTBP1 inhibited the production of ZIKV progeny (Fig. 4C). Conversely, we also constructed shPTBP1 RNA-expressing lentivirus and established shPTBP1 cells and control cells (shNC). The knockdown efficiency was confirmed by Western blot analysis (Fig. S2C). In the cells where PTBP1 was knocked down and infected with ZIKV (Fig. S2D), the levels of ZIKV RNA (Fig. 4D) and viral NS5 protein (Fig. 4E) increased significantly. In parallel, the production of ZIKV progeny was inhibited by shPTBP1 at both 24 and 48 h p.i. (Fig. 4F). Therefore, the data present that PTBP1 plays an inhibitory role in ZIKV replication.

Considering HIF-1α activation promotes PTBP1 expression, and PTBP1 inhibits ZIKV replication, we assessed whether the HIF-1α-driven PTBP1 upregulation possesses an antiviral effect on ZIKV. Then, A549 cells were pretreated with IOX2 at varying concentrations and then infected with ZIKV. We observed that ZIKV RNA level was decreased as the concentrations of IOX2 increased in shNC cells (Fig. 4G), suggesting HIF-1α activation inhibited ZIKV replication. However, IOX2-inhibited ZIKV replication was restored in shPTBP1 cells (Fig. 4G). Parallelly, IOX2 induced HIF-1α and PTBP1 expression, resulting in the inhibition of ZIKV replication in shNC cells (Fig. 4H), whereas such inhibitory role on ZIKV viral protein (Fig. 4H) and progeny production (Fig. 4I) was attenuated in shPTBP1 cells. Therefore, PTBP1 plays an inhibitory role in ZIKV replication driven by HIF-1α.

### Cytoplasm localization of PTBP1 mediates an inhibitory role on ZIKV replication

**Fig 5:**
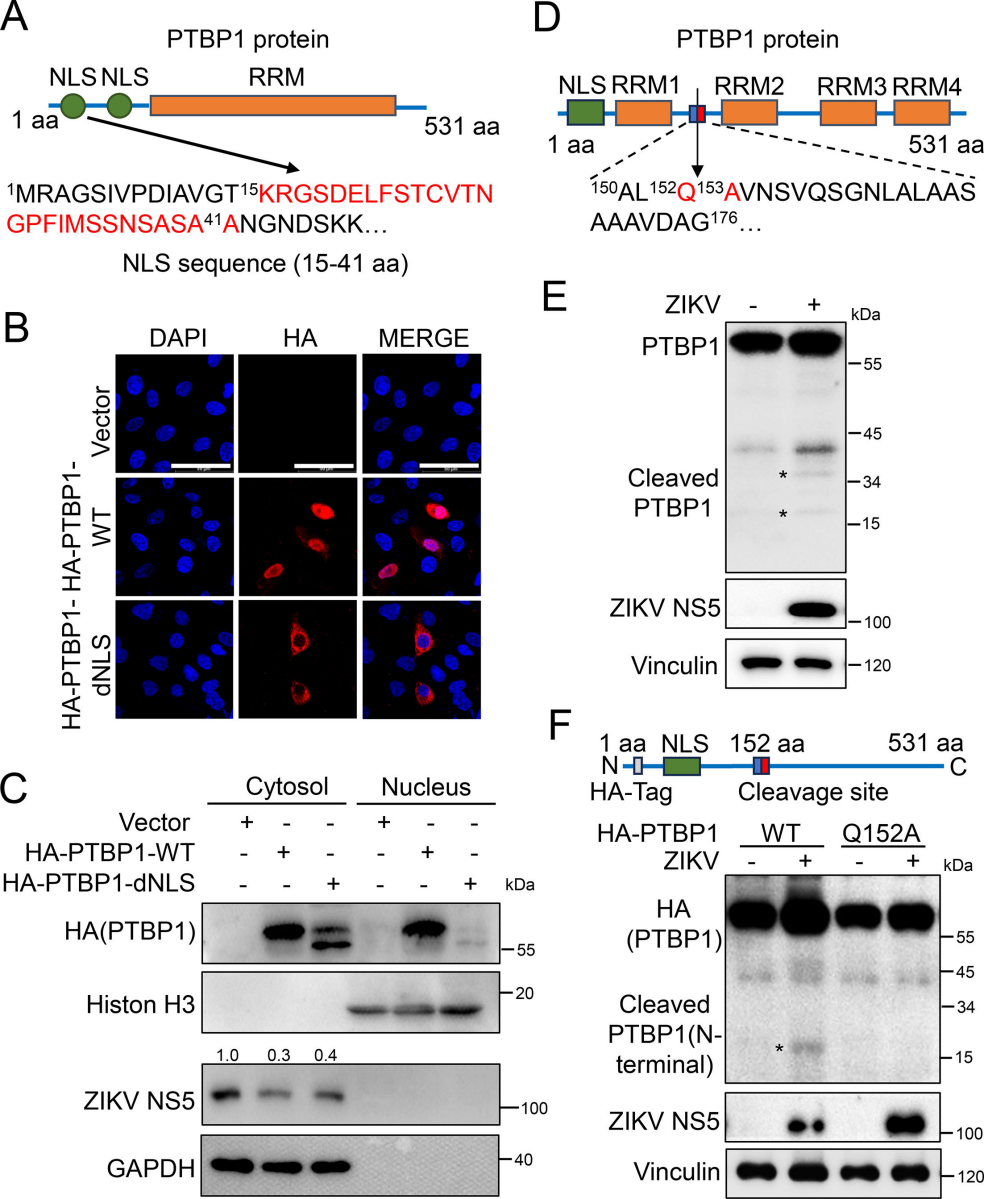
The translocation of PTBP1 upon ZIKV infection. (**A**) Schematic diagram of PTBP1 structure. NLS, nuclear localization signal; RRM, RNA recognition motif. The indicated NLS sequence is from Lys15 to Ala41. (**B and C**) A549 cells were transfected with 1.0 μg of each plasmid: HA-Vector, HA-PTBP1-WT, or HA-PTBP1-dNLS. At 24 h post-transfection, the cells’ nucleus and cytosols were subjected to immunofluorescence assay (**B**) and Western blot analysis (**C**), respectively. Scale bar = 50 µm. (**D**) Schematic diagram of the cleavage site of PTBP1 protein. (**E**) A549 cells were infected with ZIKV (MOI = 2) for 24 h, and then the cells were harvested for protein detection by Western blot analysis with primary antibodies against PTBP1 and ZIKV NS5. Vinculin is used as an internal reference. (**F**) The plasmid expressing the mutant PTBP1 protein at the cleavage site Q153A was constructed. The plasmids HA-PTBP1-WT (2.0 μg) or HA-PTBP1-Q152A (2.0 μg) were transfected into A549 cells. At 24 h post-transfection, the cells were further infected with ZIKV (MOI = 2) for an additional 24 h. The protein in cell lysis was subjected to Western blot analysis. *, cleaved PTBP1.

PTBP1 is a member of the hnRNP family that is distributed in the nucleus and cytoplasm (29), we first examined whether the inhibitory role on ZIKV replication of PTBP1 is dependent on its nuclear or cytoplasm localization. We identified 27 amino acid residues ranging from Lys15 to Ala41 as the nuclear localization signal sequence of PTBP1 by using cNLS Mapper software (Fig. 5A), which is consistent with the previous report (30). Then, we constructed an NLS (Lys15 to Ala41) deletion of PTBP1 (PTBP1-dNLS) expressing plasmid and then transfected the plasmid into A549 cells. Immunofluorescence assay exhibited both the nuclear and cytoplasm localization of full-length PTBP1 (PTBP1-WT) protein, while the cytoplasm localization of PTBP1-dNLS protein (Fig. 5B), suggesting an effective deletion of the nuclear localization signal of PTBP1. Next, the localization of PTBP1 on the inhibition of ZIKV replication was verified. The plasmids expressing PTBP1-WT or PTBP1-dNLS were transfected into A549 cells and infected with ZIKV. The harvested cells were subjected to the separation of the nucleus and cytoplasm, which was followed by Western blot analysis. We observed that the nucleus marker Histone H3 and cytoplasm marker GAPDH were clearly isolated; PTBP1-WT was distributed in both nucleus and cytoplasm, while PTBP1-dNLS was nearly totally distributed in cytoplasm (Fig. 5C). Importantly, ZIKV NS5 protein expression was inhibited in the presence of PTBP1-WT, and such inhibition still remained in the presence of PTBP1-dNLS (Fig. 5C), suggesting the inhibitory role of PTBP1 on ZIKV replication is probably dependent on its cytoplasmic localization.

To elucidate the translocation of PTBP1 cytoplasm upon ZIKV infection, we guessed whether the NLS of PTBP1 was cleaved by ZIKV. Since there is a cleavage site at Gln152/Ala153 residues in the full-length PTBP1 protein (31), the cleavage of the N-terminal of PTBP1 containing NLS upon ZIKV infection was verified (Fig. 5D). In the ZIKV-infected cells, the expression of full-length PTBP1 was increased compared to that in mock-infected cells. In addition, ZIKV infection promoted the cleavage of PTBP1 to generate proteolytic fragments (Fig. 5E), suggesting ZIKV infection could cleave PTBP1 as a substrate. To further confirm this cleavage of PTBP1 by ZIKV infection, we constructed a mutant plasmid at Q152A expressing PTBP1 (Fig. 5E, upper). The WT and mutant plasmids expressing the PTBP1 protein were transfected into A549 cells, followed by ZIKV infection. The N-terminal of PTBP1 covering HA-Tag and NLS from the cleavage of WT PTBP1 protein was generated but not detected from Q152A PTBP1 protein (Fig. 5E, lower). Thus, we concluded that the NLS of PTBP1 was cleaved by ZIKV, which contributes to the translocation of PTBP1 to cytoplasm upon ZIKV infection to exert an inhibitory effect on viral replication.

### PTBP1 interacts with ZIKV NS1

**Fig 6:**
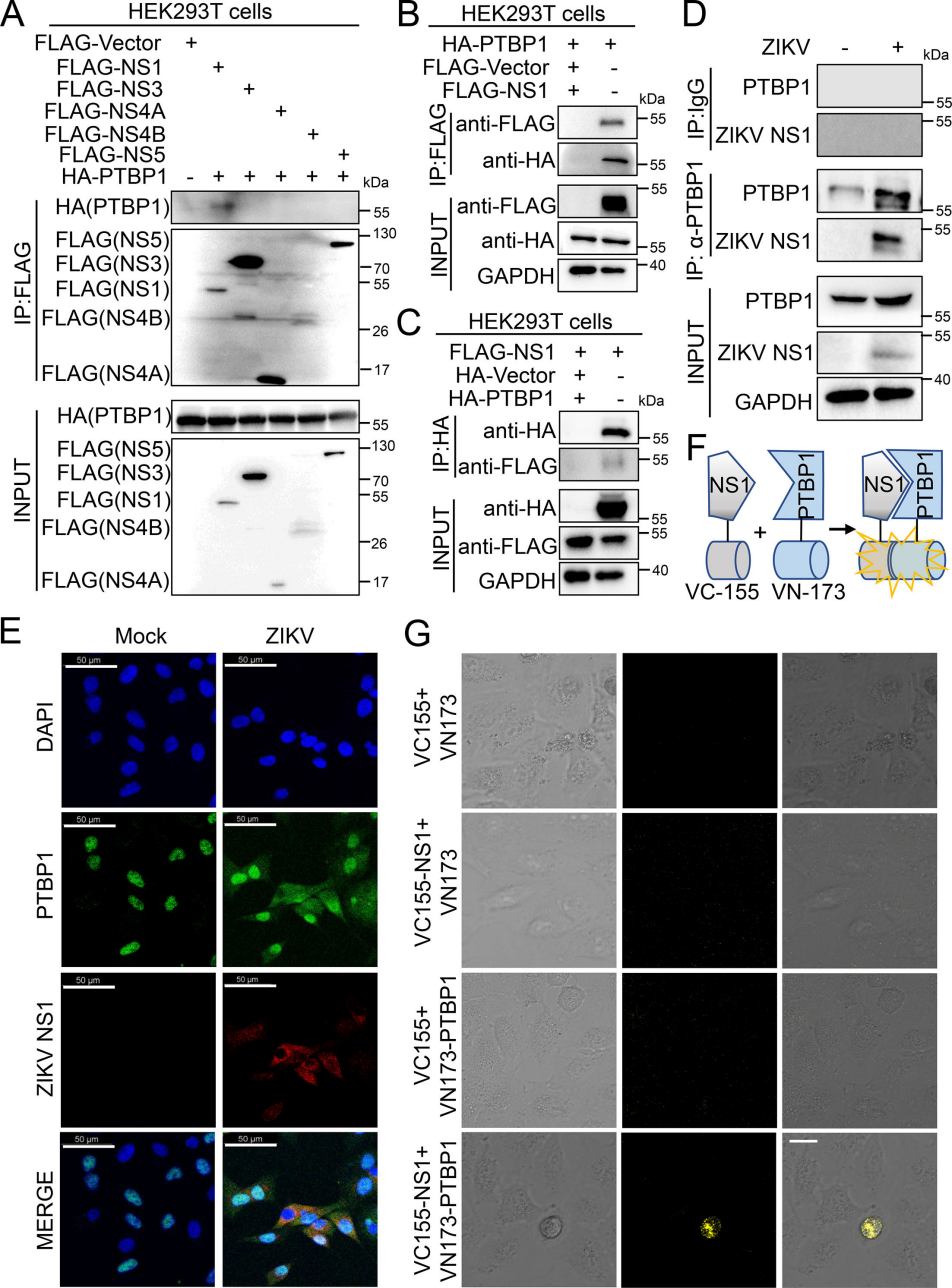
PTBP1 binds to ZIKV NS1. (**A–C**) HEK293T cells were transfected with the corresponding plasmids and harvested after 30 h. The cell lysates were incubated with anti-FLAG (**A and B**) or anti-HA (**C**) primary antibody overnight to immunoprecipitate target proteins, followed by Western blot analysis. (**D**) A549 cells were infected with ZIKV (MOI = 2) for 24 h and then harvested. The cell lysates were immunoprecipitated with anti-PTBP1 antibody, followed by Western blot analysis with the indicated antibodies. (**E**) A549 cells were infected with ZIKV (MOI = 2) for 24 h and then incubated with primary antibodies against PTBP1 and ZIKV NS1. The treated cells were labeled with corresponding fluorescent secondary antibodies and examined under a confocal fluorescence microscope. The subcellular distribution of PTBP1 (green) and ZIKV NS1 (red), along with the nucleus stained with DAPI (blue), was captured. Scale bar = 50 µm. (**F**) Schematic of bimolecular fluorescence complementation (BiFc) assay. (**G**) A549 cells were co-transfected with Venus BiFc plasmids expressing control protein (VC155 or VN173), NS1 protein (VC155-NS1), and PTBP1 protein (VN173-PTBP1), respectively. At 24 h post-transfection, the fluorescence was visualized by confocal microscopy. Scale bar = 20 µm.

To delve into the exact mechanisms by which PTBP1 inhibits ZIKV infection, we focused on the relationship between PTBP1 and the non-structural proteins of ZIKV. We co-transfected the HA-PTBP1 plasmid with an individual plasmid encoding FLAG-tagged ZIKV non-structural proteins, including FLAG-NS1, FLAG-NS3, FLAG-NS4A, FLAG-NS4B, and FLAG-NS5, followed by conducting Co-IP experiments in HEK293T cells. The results demonstrated that PTBP1 was specifically immunoprecipitated with the ZIKV NS1 protein (Fig. 6A), suggesting that PTBP1 binds to viral NS1 protein. To further validate this interaction, we performed exogenous proteins by Co-IP assays in HEK293T cells after co-transfecting HA-PTBP1 and FLAG-NS1 plasmids. In pull-down assays using FLAG (Fig. 6B) or HA (Fig. 6C) antibodies, respectively, we affirmed the interaction between PTBP1 and ZIKV NS1.

Next, we verified the interaction between two endogenous proteins in ZIKV-infected A549 cells. The PTBP1 antibody immunoprecipitated endogenous PTBP1 and NS1 in ZIKV-infected cells (Fig. 6D). In the immunofluorescence assay, we observed the intracellular localization of PTBP1 in the nucleus in the mock-infected cells, whereas PTBP1 was mainly distributed in the cytoplasm in cells where the ZIKV replication occurred (Fig. 6E). Importantly, the PTBP1 in the cytoplasm strongly colocalized with ZIKV NS1 (Fig. 6E), indicating the interaction between endogenous PTBP1 and NS1. To further verify the physical interaction of ZIKV NS1 and PTBP1, the BiFc assay was employed in the living cell (Fig. 6F), which reflects the direct interaction between the two fusion proteins with the complementary yellow fluorescence protein (YFP). In the absence of either VC155-NS1 or VN173-PTBP1, the YFP in cells failed to be observed as expected (Fig. 6G), while in the presence of VC155-NS1 and VN173-PTBP1, the YFP in cells was observed (Fig. 6G), suggesting a strong physical interaction between NS1 and PTBP1 proteins. Thus, these data reveal that PTBP1 interacts with ZIKV NS1 to inhibit viral replication.

### PTBP1 induces the degradation of ZIKV NS1 protein through the lysosomal pathway

**Fig 7:**
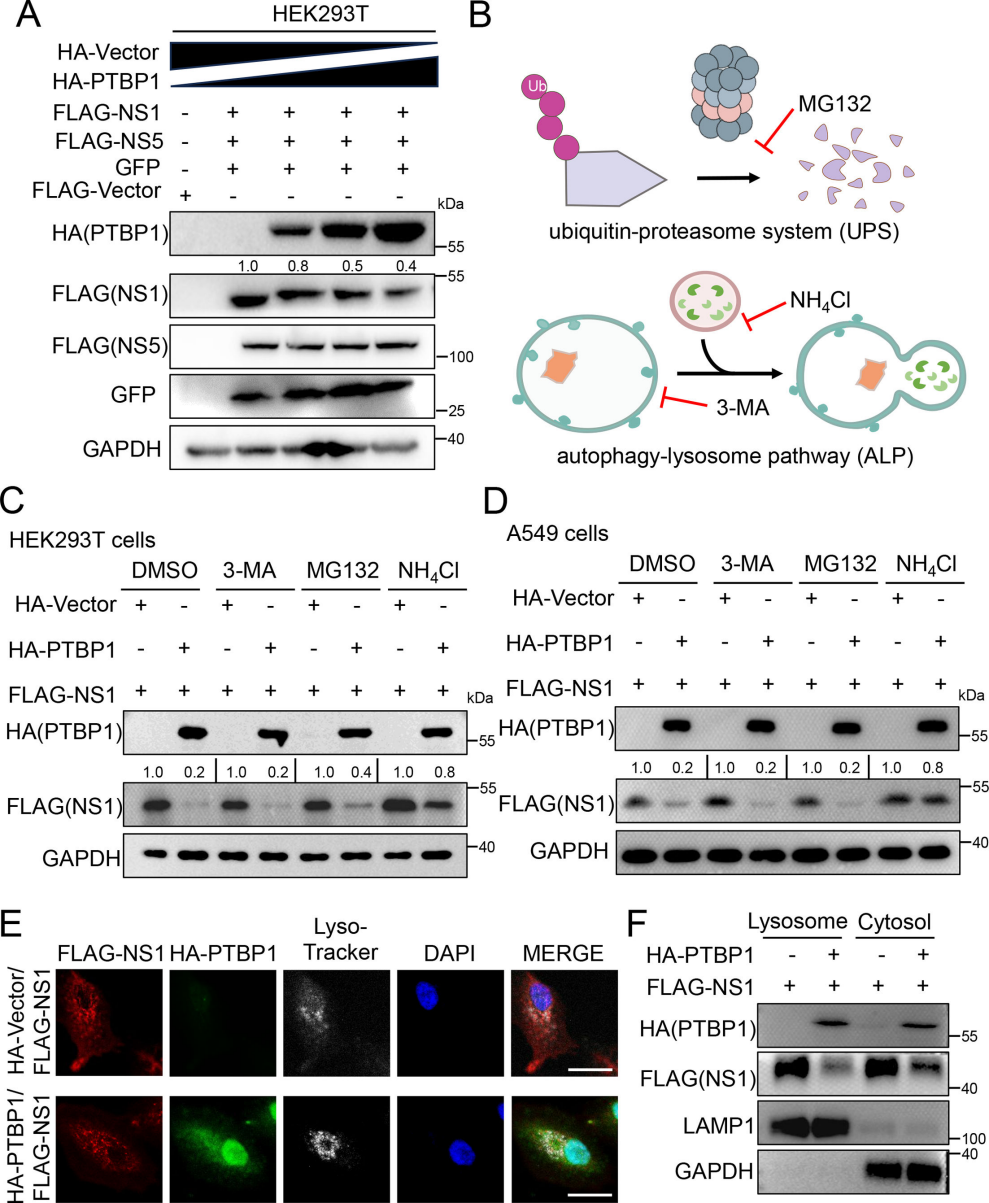
PTBP1 induces the degradation of ZIKV NS1 protein through the lysosomal pathway. (**A**) HEK293T cells were transfected with the HA-Vector (2.0, 1.75, 1.5, 1.25, and 0.75 μg) and FLAG-Vector (1.0, 0, 0, 0, 0 μg) or FLAG-NS1 (0, 0, 0, 0, 0.5 μg), or FLAG-NS5 (0, 0, 0, 0, 0.5 μg) or HA-PTBP1 (0, 0, 0.25, 0.5, 1.0) along with eGFP-C1 (0, 0.25, 0.25, 0.25, 0.25 μg) plasmids. After 30 h, the cells were collected and subjected to Western blot analysis. (**B**) The scheme of two main models of protein degradation: UPS (ubiquitin-proteasome system) and ALP (autophagy-lysosome pathway). (**C and D**) HEK293T cells (**C**) or A549 cells (**D**) were transfected with 1.5 μg of each plasmid: HA-Vector, FLAG-NS1, and HA-PTBP1, along with FLAG-NS1. At 18 h post-transfection, the cells were treated with DMSO, 3-MA (1.0 mM), MG132 (2.5 μM), or NH_4_Cl (5 mM) for another 12 h. The protein levels were analyzed by Western blot. (**E**) A549 cells were transfected with 1.0 μg of each plasmid: HA-Vector or HA-PTBP1, along with FLAG-NS1. At 24 h post-transfection, the cells were labeled with anti-FLAG and anti-HA primary antibodies followed by corresponding fluorescent secondary antibodies. The lysosomes were stained with Lyso-Tracker, while the nucleus was stained with DAPI. The images displaying NS1 (red), PTBP1 (green), Lysosome (white), and DAPI (blue) were captured under a confocal fluorescence microscope. Scale bar = 20 µm. (**F**) A549 cells were transfected with 1.0 μg of each plasmid: HA-Vector or HA-PTBP1 along with FLAG-NS1. At 24 h post-transfection, the cells were harvested, and lysosome isolation was performed. The cell’s lysosomal and the rest of the cytosol fractions were subjected to Western blot analysis.

To get a full understanding of how PTBP1 interacts with the ZIKV NS1 protein to inhibit viral replication, we investigated the impact of PTBP1 on the stability of the ZIKV NS1 protein. In the presence of the increasing amount of the HA-PTBP1, the expression level of ZIKV NS1 protein was significantly reduced, while the expression level of either ZIKV NS5 or GFP protein remained unchanged (Fig. 7A), indicating that PTBP1 specifically degrades the ZIKV NS1 protein.

Given that the protein degradation belongs to two main models: UPS (ubiquitin-proteasome system) and ALP (autophagy-lysosome pathway) (Fig. 7B), we applied autophagy inhibitor 3-MA, proteasome inhibitor MG132, and lysosomal inhibitor NH_4_Cl to treat cells co-expressing PTBP1 and NS1. In HEK293T cells, PTBP1 reduced NS1 protein level; however, the NH_4_Cl restored the degradation of ZIKV NS1 protein induced by PTBP1, while 3-MA and MG132 had no such effects (Fig. 7C). In A549 cells, we also observed similar results (Fig. 7D), suggesting PTBP1 induced the degradation of the ZIKV NS1 protein through the lysosomal pathway. Furthermore, we observed the subcellular distribution of NS1 protein and found a certain portion of NS1 protein located in the lysosome in the absence of PTBP1 (Fig. 7E). Nevertheless, the portion of NS1 protein located in the lysosome was eliminated in the presence of PTBP1 (Fig. 7E). To confirm the interplay of PTBP1 ZIKV NS1 in the lysosome, we perform the isolation of lysosome from cytoplasm and Western blot analysis. The harvested cells transfected with PTBP1 and NS1 were separated into lysosome and cytosol fractions, which were indicated by LAMP1 and GAPDH (Fig. 7F), respectively. In the presence of PTBP1, NS1 expression was reduced in cytosol, whereas the reduction of NS1 was enhanced in the lysosome (Fig. 7F), suggesting a degradation of ZIKV NS1 protein by PTBP1 in lysosome. Together, these results illustrate that PTBP1 induces the degradation of ZIKV NS1 protein via the lysosomal pathway.

Since degradation of NS1 can directly affect ZIKV replication, there is another possibility that the degradation of NS1 affects TANK-binding kinase 1 (TBK1) activation (phosphorylation), leading to the innate immunity-mediated viral suppression (32). To this end, we investigated the degradation of NS1 mediated by PTBP1 on the TBK1 activation. In the presence of PTBP1, the degradation of NS1 was observed, while the TBK1 phosphorylation was enhanced (Fig. S3), suggesting PTBP1 mediated the degradation of NS1 to promote TBK1 activation and antiviral immunity. Together, PTBP1 binds ZIKV NS1 protein and degrades NS1, which directly disturbs viral replication and also enhances antiviral immunity, resulting in the suppression of ZIKV replication.

**Fig 8:**
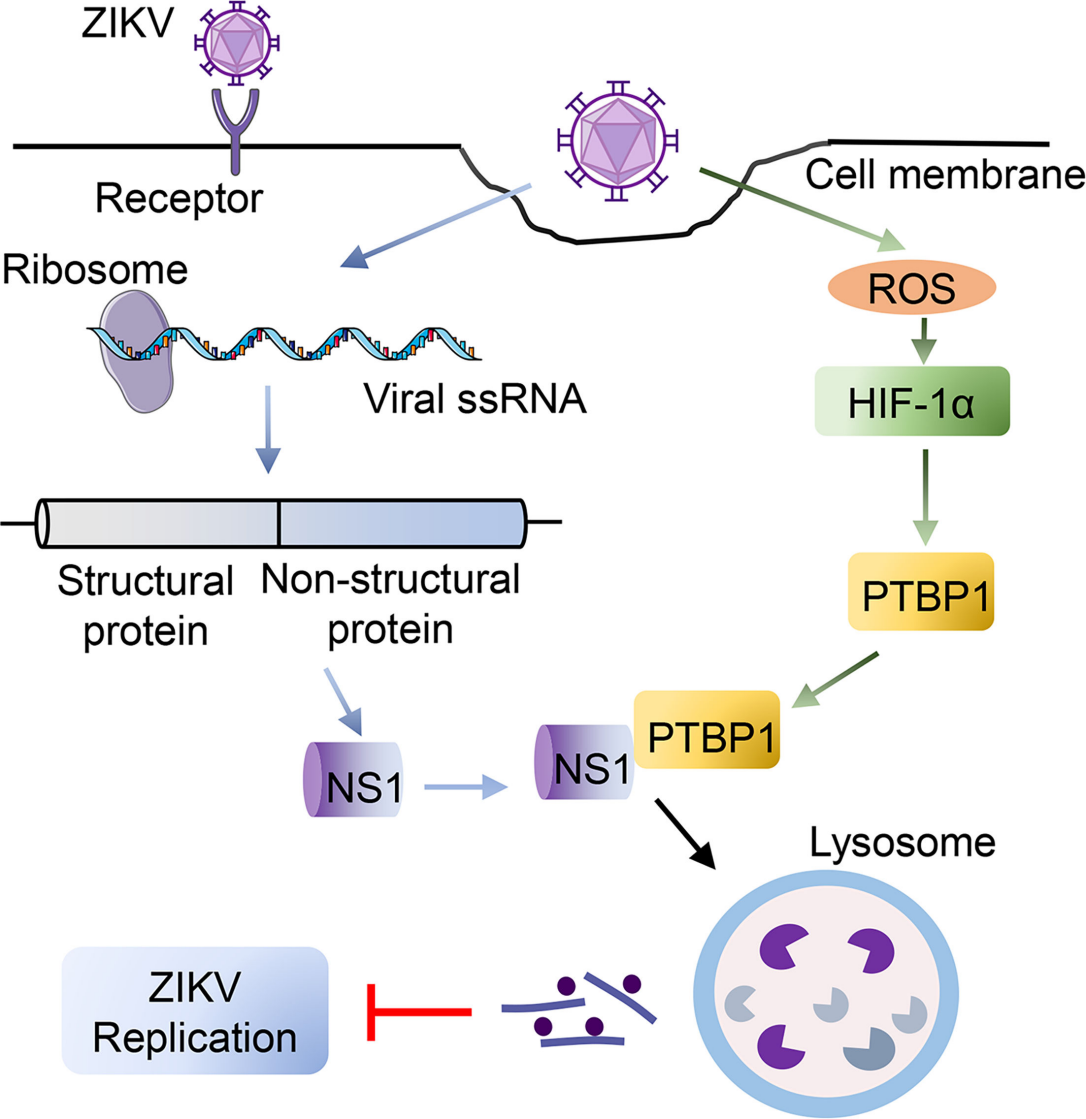
Proposal model underlying PTBP1 interacts with ZIKV NS1 to inhibit viral replication. Upon ZIKV infection, ROS is initiated, and the HIF-1α signal is activated to promote PTBP1 expression. Meanwhile, ZIKV viral RNA is translated into structural and nonstructural proteins, which participate in viral replication. The HIF-1α-induced PTBP1 interacts with ZIKV NS1 and then promotes the degradation of the NS1 protein in a lysosomal pathway and finally disrupts ZIKV replication.

In sum, our findings exhibited that ZIKV infection induces the expression of PTBP1 via the ROS-HIF-1α pathway; in turn, the PTBP1 binds ZIKV NS1 protein to promote NS1 degradation, thereby effectively inhibiting viral replication (Fig. 8).

## DISCUSSION

ZIKV has previously triggered large-scale outbreaks globally, posing a severe threat to public health. However, to date, no effective vaccine or treatment is available. PTBP1 is an RNA-binding protein with a crucial role, and in recent years, its potential in inhibiting the replication of *Flaviviridae* viruses has gradually been unveiled. Specifically, Deepika et al. have shown that during Japanese encephalitis virus (JEV) infection, PTBP1 can translocate to the cytoplasm and effectively inhibit viral replication by competitively inhibiting the binding of JEV RNA to the NS5 protein (33). Jiang et al. have found that PTBP1 can bind to the NS4A protein and RNA of dengue virus (DENV), affecting virus production by regulating the synthesis of negative-strand RNA (34). The role of PTBP1 on ZIKV replication raises new insight into the interaction between PTBP1 and *Flaviviridae* viruses.

In our study, we observed that ZIKV infection significantly upregulates the expression level of PTBP1, a process that involves the activation of HIF-1α. In hypoxic environments, the expression of HIF-1α stabilizes and combines with HIF-1β to form HIFs, which regulate downstream signaling pathways and gene expression activity, thereby modulating various cellular adaptations and inflammatory responses (35, 36). Further studies indicate that modulating the expression level of PTBP1 directly restrains ZIKV replication. Specifically, HIF-1α induced-PTBP1 inhibits ZIKV replication, suggesting the activation of HIF-1α inhibits viral replication. These findings further confirm the significant role of PTBP1 in the antiviral effect. In our previous finding, we reveal that severe acute respiratory syndrome coronavirus 2 (SARS-CoV-2) infection induces HIF-1α expression to aggravate inflammatory responses to Coronavirus disease 2019 (COVID-19) (37). Notably, HIF-1α activation promotes SARS-CoV-2 replication, which differs from this study that HIF-1α activation inhibits ZIKV replication by induction of PTBP1. As such, the control of HIF signaling is thought to be a promising prospect for therapeutics in infectious diseases (38), including ZIKV infection and associated disorders.

Numerous studies have revealed that the interaction between host proteins and the non-structural proteins of ZIKV is one of the key mechanisms for inhibiting viral infection. For instance, the interferon-induced protein Viperin can specifically target the ZIKV NS3 and induce its degradation through the proteasome pathway, thereby effectively inhibiting viral replication (39). Similarly, the splicing factor Splicing factor 3b subunit 3 (SF3B3) protein can bind to the ZIKV NS5 and restrict viral replication by regulating GTP cyclohydroxylase 1 (GCH1) (40). Here, we explore the specific mechanism by which PTBP1 exerts its antiviral effect. Furthermore, PTBP1 can induce the degradation of ZIKV NS1. We validated the specific binding of PTBP1 and ZIKV NS1 using Co-IP and immunofluorescence approaches and determined their primary localization in the cytoplasm. PTBP1 interacts with the ZIKV NS1 to inhibit viral replication. The discovery of this new manner provides important theoretical support for the crucial role of PTBP1 in the antiviral process of ZIKV.

In eukaryotic cells, protein degradation is primarily accomplished through the ubiquitin-proteasome system and the autophagy-lysosome pathway, both of which play vital roles in maintaining intracellular homeostasis and protein quality control (41, 42). Previous studies have enlightened us with some clues. For example, ZIKV NS1 and ZIKV NS3 proteins have been found to bind to Poly (ADP-Ribose) Polymerase Family Member 12 (PARP12) and are degraded via the ubiquitin-proteasome pathway (43). In addition, C19orf66 has been shown to induce the degradation of ZIKV NS3 through a lysosome-dependent pathway (44). These findings suggest that PTBP1 may induce the degradation of ZIKV NS1 through a similar mechanism. To elucidate this issue, specific inhibitors are used to block the ubiquitin-proteasome system or the autophagy-lysosome pathway, respectively, and we observed that PTBP1-induced degradation of ZIKV NS1 is dependent on the lysosome pathway. This reveals a novel mechanism by which PTBP1 inhibits viral replication by specifically binding to and inducing the degradation of ZIKV NS1. These discoveries provide important insights into the understanding of the ZIKV replication process and the development of new therapeutic strategies.

In conclusion, this study reveals that ZIKV infection upregulates the expression level of PTBP1 by activating HIF-1α. PTBP1 exhibits a significant inhibitory effect on the replication process of ZIKV. In addition, PTBP1 can interact with ZIKV NS1 protein and induce its degradation through the lysosomal pathway, thereby inhibiting viral replication. This discovery provides a potential target for viral replication and pathogenesis in ZIKV-associated diseases.

## Data Availability

All relevant data are available from the corresponding author upon request.

## SUPPLEMENTAL MATERIAL

Figures S1 to S3 jvi.01495-25-s0001.docx Fig. S1: HIF-1α activation promotes ZIKV-induced PTBP1 expression. Fig. S2: The expression of PTBP1 in PTBP1 -overexpressing and -knocking-down cells. Fig. S3: PTBP1-mediated ZIKV NS1 degradation enhances TBK1 activation.

ASM does not own the copyrights to Supplemental Material that may be linked to, or accessed through, an article. The authors have granted ASM a non-exclusive, world-wide license to publish the Supplemental Material files. Please contact the corresponding author directly for reuse.

## References

[1] Musso D, Gubler DJ. 2016. Zika Virus. Clin Microbiol Rev 29:487–524. doi:10.1128/CMR.00072-15 27029595 PMC4861986

[2] Pierson TC, Diamond MS. 2018. The emergence of Zika virus and its new clinical syndromes. Nature 560:573–581. doi:10.1038/s41586-018-0446-y 30158602

[3] Zorrilla CD, García García I, García Fragoso L, De La Vega A. 2017. Zika virus infection in pregnancy: maternal, fetal, and neonatal considerations. J Infect Dis 216:S891–S896. doi:10.1093/infdis/jix448 29267916 PMC5853951

[4] Kassavetis P, Joseph J-MB, Francois R, Perloff MD, Berkowitz AL. 2016. Zika virus-associated Guillain-Barré syndrome variant in Haiti. Neurology 87:336–337. doi:10.1212/WNL.0000000000002759 27164708

[5] Mancera-Páez O, Román GC, Pardo-Turriago R, Rodríguez Y, Anaya JM. 2018. Concurrent Guillain-Barré syndrome, transverse myelitis and encephalitis post-Zika: A case report and review of the pathogenic role of multiple arboviral immunity. J Neurol Sci 395:47–53. doi:10.1016/j.jns.2018.09.028 30292020

[6] Lin HH, Yip BS, Huang LM, Wu SC. 2018. Zika virus structural biology and progress in vaccine development. Biotechnol Adv 36:47–53. doi:10.1016/j.biotechadv.2017.09.004 28916391

[7] Beckham JD, Pastula DM, Massey A, Tyler KL. 2016. Zika virus as an emerging global pathogen: neurological complications of Zika virus. JAMA Neurol 73:875–879. doi:10.1001/jamaneurol.2016.0800 27183312 PMC5087605

[8] Rice CM, Lenches EM, Eddy SR, Shin SJ, Sheets RL, Strauss JH. 1985. Nucleotide sequence of yellow fever virus: implications for Flavivirus gene expression and evolution. Science 229:726–733. doi:10.1126/science.4023707 4023707

[9] Ávila-Pérez G, Nogales A, Martín V, Almazán F, Martínez-Sobrido L. 2018. Reverse genetic approaches for the generation of recombinant Zika virus. Viruses 10:597. doi:10.3390/v10110597 30384426 PMC6266887

[10] Hu H, Feng Y, He ML. 2023. Targeting type I interferon induction and signaling: how Zika virus escapes from host innate immunity. Int J Biol Sci 19:3015–3028. doi:10.7150/ijbs.83056 37416780 PMC10321277

[11] Shi Y, Dai L, Song H, Gao GF. 2018. Structures of Zika virus E & NS1: relations with virus infection and host immune responses. Adv Exp Med Biol 1062:77–87. doi:10.1007/978-981-10-8727-1_6 29845526

[12] Wu Y, Liu Q, Zhou J, Xie W, Chen C, Wang Z, Yang H, Cui J. 2017. Zika virus evades interferon-mediated antiviral response through the co-operation of multiple nonstructural proteins in vitro. Cell Discov 3:17006. doi:10.1038/celldisc.2017.6 28373913 PMC5359216

[13] Zheng Y, Liu Q, Wu Y, Ma L, Zhang Z, Liu T, Jin S, She Y, Li YP, Cui J. 2018. Zika virus elicits inflammation to evade antiviral response by cleaving cGAS via NS1-caspase-1 axis. EMBO J 37:e99347. doi:10.15252/embj.201899347 30065070 PMC6138430

[14] Bailey MJ, Broecker F, Duehr J, Arumemi F, Krammer F, Palese P, Tan GS. 2019. Antibodies elicited by an NS1-based vaccine protect mice against Zika virus. MBio 10:e02861-18. doi:10.1128/mBio.02861-18 30940710 PMC6445944

[15] Oberstrass FC, Auweter SD, Erat M, Hargous Y, Henning A, Wenter P, Reymond L, Amir-Ahmady B, Pitsch S, Black DL, Allain FH-T. 2005. Structure of PTB bound to RNA: specific binding and implications for splicing regulation. Science 309:2054–2057. doi:10.1126/science.1114066 16179478

[16] Conte MR, Grüne T, Ghuman J, Kelly G, Ladas A, Matthews S, Curry S. 2000. Structure of tandem RNA recognition motifs from polypyrimidine tract binding protein reveals novel features of the RRM fold. EMBO J 19:3132–3141. doi:10.1093/emboj/19.12.3132 10856256 PMC203357

[17] Chen XD, Liu HL, Li S, Hu KB, Wu QY, Liao P, Wang HY, Long ZY, Lu XM, Wang YT. 2023. The latest role of nerve-specific splicing factor PTBP1 in the transdifferentiation of glial cells into neurons. Wiley Interdiscip Rev RNA 14:e1740. doi:10.1002/wrna.1740 35574699

[18] Romanelli MG, Diani E, Lievens PM-J. 2013. New insights into functional roles of the polypyrimidine tract-binding protein. Int J Mol Sci 14:22906–22932. doi:10.3390/ijms141122906 24264039 PMC3856098

[19] Mitchell SA, Brown EC, Coldwell MJ, Jackson RJ, Willis AE. 2001. Protein factor requirements of the Apaf-1 internal ribosome entry segment: roles of polypyrimidine tract binding protein and upstream of N-ras. Mol Cell Biol 21:3364–3374. doi:10.1128/MCB.21.10.3364-3374.2001 11313462 PMC100258

[20] Kim W, Shin JC, Lee KH, Kim KT. 2020. PTBP1 positively regulates the translation of circadian clock gene, Period1. Int J Mol Sci 21:6921. doi:10.3390/ijms21186921 32967200 PMC7555454

[21] Shereen MA, Bashir N, Su R, Liu F, Wu K, Luo Z, Wu J. 2021. Zika virus dysregulates the expression of astrocytic genes involved in neurodevelopment. PLoS Negl Trop Dis 15:e0009362. doi:10.1371/journal.pntd.0009362 33891593 PMC8099136

[22] Wang W, Li G, Luo Z, Pan P, Tian M, Wang Y, Xiao F, Li A, Wu K, Liu X, Rao L, Liu F, Liu Y, Wu J, De W. 2018. Zika virus infection induces host inflammatory responses by facilitating NLRP3 inflammasome assembly and interleukin-1β secretion. Nat Commun 9:106. doi:10.1038/s41467-017-02645-3 29317641 PMC5760693

[23] Chen W, Li Y, Yu X, Wang Z, Wang W, Rao M, Li Y, Luo Z, Zhang Q, Liu J, Wu J. 2023. Zika virus non-structural protein 4B interacts with DHCR7 to facilitate viral infection. Virol Sin 38:23–33. doi:10.1016/j.virs.2022.09.009 36182074 PMC10006206

[24] Luo Z, Ge M, Chen J, Geng Q, Tian M, Qiao Z, Bai L, Zhang Q, Zhu C, Xiong Y, Wu K, Liu F, Liu Y, Wu J. 2017. HRS plays an important role for TLR7 signaling to orchestrate inflammation and innate immunity upon EV71 infection. PLoS Pathog 13:e1006585. doi:10.1371/journal.ppat.1006585 28854257 PMC5595348

[25] Ruan Z, Liang Y, Chen Z, Yin J, Li C, Pan P, Zhang Q, Wu J, Luo Z. 2022. Enterovirus 71 non-structural protein 3A hijacks vacuolar protein sorting 25 to boost exosome biogenesis to facilitate viral replication. Front Microbiol 13:1024899. doi:10.3389/fmicb.2022.1024899 36274707 PMC9581156

[26] Schouest B, Peterson TA, Szeltner DM, Scheef EA, Baddoo M, Ungerleider N, Flemington EK, MacLean AG, Maness NJ. 2021. Transcriptional signatures of Zika virus infection in astrocytes. J Neurovirol 27:116–125. doi:10.1007/s13365-020-00931-3 33405202 PMC7921019

[27] Dery KJ, Kojima H, Kageyama S, Kadono K, Hirao H, Cheng B, Zhai Y, Farmer DG, Kaldas FM, Yuan X, Eltzschig HK, Kupiec-Weglinski JW. 2023. Alternative splicing of CEACAM1 by hypoxia-inducible factor-1α enhances tolerance to hepatic ischemia in mice and humans. Sci Transl Med 15:eadf2059. doi:10.1126/scitranslmed.adf2059 37531413 PMC11164245

[28] Dong S, Liang S, Cheng Z, Zhang X, Luo L, Li L, Zhang W, Li S, Xu Q, Zhong M, Zhu J, Zhang G, Hu S. 2022. ROS/PI3K/Akt and Wnt/β-catenin signalings activate HIF-1α-induced metabolic reprogramming to impart 5-fluorouracil resistance in colorectal cancer. J Exp Clin Cancer Res 41:15. doi:10.1186/s13046-021-02229-6 34998404 PMC8742403

[29] Alber S, Di Matteo P, Zdradzinski MD, Dalla Costa I, Medzihradszky KF, Kawaguchi R, Di Pizio A, Freund P, Panayotis N, Marvaldi L, Doron-Mandel E, Okladnikov N, Rishal I, Nevo R, Coppola G, Lee SJ, Sahoo PK, Burlingame AL, Twiss JL, Fainzilber M. 2023. PTBP1 regulates injury responses and sensory pathways in adult peripheral neurons. Sci Adv 9:eadi0286. doi:10.1126/sciadv.adi0286 37506203 PMC10381954

[30] Desideri F, Cipriano A, Petrezselyova S, Buonaiuto G, Santini T, Kasparek P, Prochazka J, Janson G, Paiardini A, Calicchio A, Colantoni A, Sedlacek R, Bozzoni I, Ballarino M. 2020. Intronic determinants coordinate charme lncRNA nuclear activity through the interaction with MATR3 and PTBP1. Cell Rep 33:108548. doi:10.1016/j.celrep.2020.108548 33357424 PMC7773549

[31] Pablos I, Machado Y, de Jesus HCR, Mohamud Y, Kappelhoff R, Lindskog C, Vlok M, Bell PA, Butler GS, Grin PM, et al.. 2021. Mechanistic insights into COVID-19 by global analysis of the SARS-CoV-2 3CL^pro^ substrate degradome. Cell Rep 37:109892. doi:10.1016/j.celrep.2021.109892 34672947 PMC8501228

[32] Xia H, Luo H, Shan C, Muruato AE, Nunes BTD, Medeiros DBA, Zou J, Xie X, Giraldo MI, Vasconcelos PFC, Weaver SC, Wang T, Rajsbaum R, Shi PY. 2018. An evolutionary NS1 mutation enhances Zika virus evasion of host interferon induction. Nat Commun 9:414. doi:10.1038/s41467-017-02816-2 29379028 PMC5788864

[33] Bhullar D, Jalodia R, Kalia M, Vrati S. 2014. Cytoplasmic translocation of polypyrimidine tract-binding protein and its binding to viral RNA during Japanese encephalitis virus infection inhibits virus replication. PLoS One 9:e114931. doi:10.1371/journal.pone.0114931 25545659 PMC4278868

[34] Jiang L, Yao H, Duan X, Lu X, Liu Y. 2009. Polypyrimidine tract-binding protein influences negative strand RNA synthesis of dengue virus. Biochem Biophys Res Commun 385:187–192. doi:10.1016/j.bbrc.2009.05.036 19450550 PMC7117538

[35] Downes NL, Laham-Karam N, Kaikkonen MU, Ylä-Herttuala S. 2018. Differential but complementary HIF1α and HIF2α transcriptional regulation. Mol Ther 26:1735–1745. doi:10.1016/j.ymthe.2018.05.004 29843956 PMC6036226

[36] Lee SH, Golinska M, Griffiths JR. 2021. HIF-1-independent mechanisms regulating metabolic adaptation in hypoxic cancer cells. Cells 10:2371. doi:10.3390/cells10092371 34572020 PMC8472468

[37] Tian M, Liu W, Li X, Zhao P, Shereen MA, Zhu C, Huang S, Liu S, Yu X, Yue M, Pan P, Wang W, Li Y, Chen X, Wu K, Luo Z, Zhang Q, Wu J. 2021. HIF-1α promotes SARS-CoV-2 infection and aggravates inflammatory responses to COVID-19. Signal Transduct Target Ther 6:308. doi:10.1038/s41392-021-00726-w 34408131 PMC8371950

[38] Luo Z, Tian M, Yang G, Tan Q, Chen Y, Li G, Zhang Q, Li Y, Wan P, Wu J. 2022. Hypoxia signaling in human health and diseases: implications and prospects for therapeutics. Signal Transduct Target Ther 7:218. doi:10.1038/s41392-022-01080-1 35798726 PMC9261907

[39] Panayiotou C, Lindqvist R, Kurhade C, Vonderstein K, Pasto J, Edlund K, Upadhyay AS, Överby AK. 2018. Viperin restricts Zika virus and tick-borne encephalitis virus replication by targeting NS3 for proteasomal degradation. J Virol 92:e02054-17. doi:10.1128/JVI.02054-17 29321318 PMC5972904

[40] Chen T, Yang H, Liu P, Hamiti M, Zhang X, Xu Y, Quan W, Zhang Y, Yu W, Jiao L, Du T, Xi J, Yin B, Zhou W, Lu S, Peng X. 2023. Splicing factor SF3B3, a NS5-binding protein, restricts ZIKV infection by targeting GCH1. Virol Sin 38:222–232. doi:10.1016/j.virs.2022.12.005 36572150 PMC10176263

[41] Zhao L, Zhao J, Zhong K, Tong A, Jia D. 2022. Targeted protein degradation: mechanisms, strategies and application. Signal Transduct Target Ther 7:113. doi:10.1038/s41392-022-00966-4 35379777 PMC8977435

[42] Kwon YT, Ciechanover A. 2017. The ubiquitin code in the ubiquitin-proteasome system and autophagy. Trends Biochem Sci 42:873–886. doi:10.1016/j.tibs.2017.09.002 28947091

[43] Li L, Zhao H, Liu P, Li C, Quanquin N, Ji X, Sun N, Du P, Qin CF, Lu N, Cheng G. 2018. PARP12 suppresses Zika virus infection through PARP-dependent degradation of NS1 and NS3 viral proteins. Sci Signal 11:eaas9332. doi:10.1126/scisignal.aas9332 29921658 PMC6434931

[44] Wu Y, Yang X, Yao Z, Dong X, Zhang D, Hu Y, Zhang S, Lin J, Chen J, An S, Ye H, Zhang S, Qiu Z, He Z, Huang M, Wei G, Zhu X. 2020. C19orf66 interrupts Zika virus replication by inducing lysosomal degradation of viral NS3. PLoS Negl Trop Dis 14:e0008083. doi:10.1371/journal.pntd.0008083 32150556 PMC7082052

