# Supplementary material for: PTBP1 is upregulated in response to Zika virus infection and restrains viral replication by hijacking viral NS1 protein to induce NS1 degradation
Source: J Virol. 2026 Apr 27;100(5):e01495-25. doi: 10.1128/jvi.01495-25 (PMC13185633; doi:10.1128/jvi.01495-25)
Supplement: Figures S1 to S3 — Fig. S1: HIF-1α activation promotes ZIKV-induced PTBP1 expression. Fig. S2: The expression of PTBP1 in PTBP1 -overexpressing and -knocking-down cells. Fig. S3: PTBP1-mediated ZIKV NS1 degradation enhances TBK1 activation. [file jvi.01495-25-s0001.docx]

**Supplemental material**

**
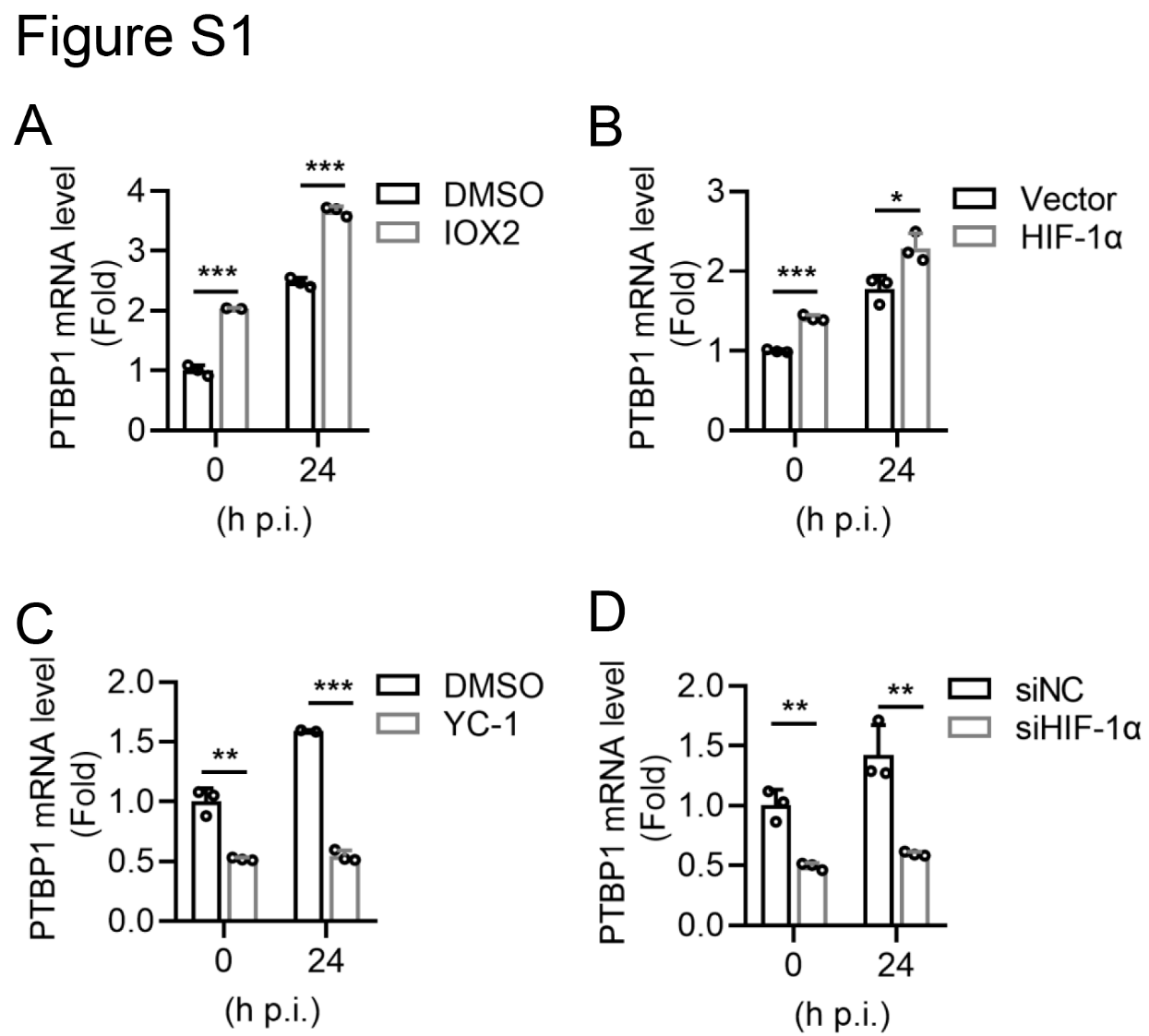
**

**Fig. S1 HIF-1α activation promotes ZIKV-induced PTBP1 expression.**

(**A** and **B**) A549 cells were transfected with pcDNA-3.1-HIF-1α-Myc-His (2 μg) (A) or stimulated with IOX2 (40 μM) (B) for 12 h, followed by infection with ZIKV (MOI = 1) for an additional 24 h. Cells were collected for qPCR analysis, respectively. (**C** and **D**) A549 cells were transfected with siRNA targeting HIF-1α (siHIF-1α) (50 nM) (C) or stimulated with a HIF-1α inhibitor YC-1 (20 μM) (D) for 16 hours, followed by infection with ZIKV (MOI = 1) for another 24 h. Cells were collected for qPCR analysis, respectively. Graphs were expressed as Mean±SD, n = 3. ns, not-significant; *, P < 0.05; **, P < 0.01; ***, P < 0.001.


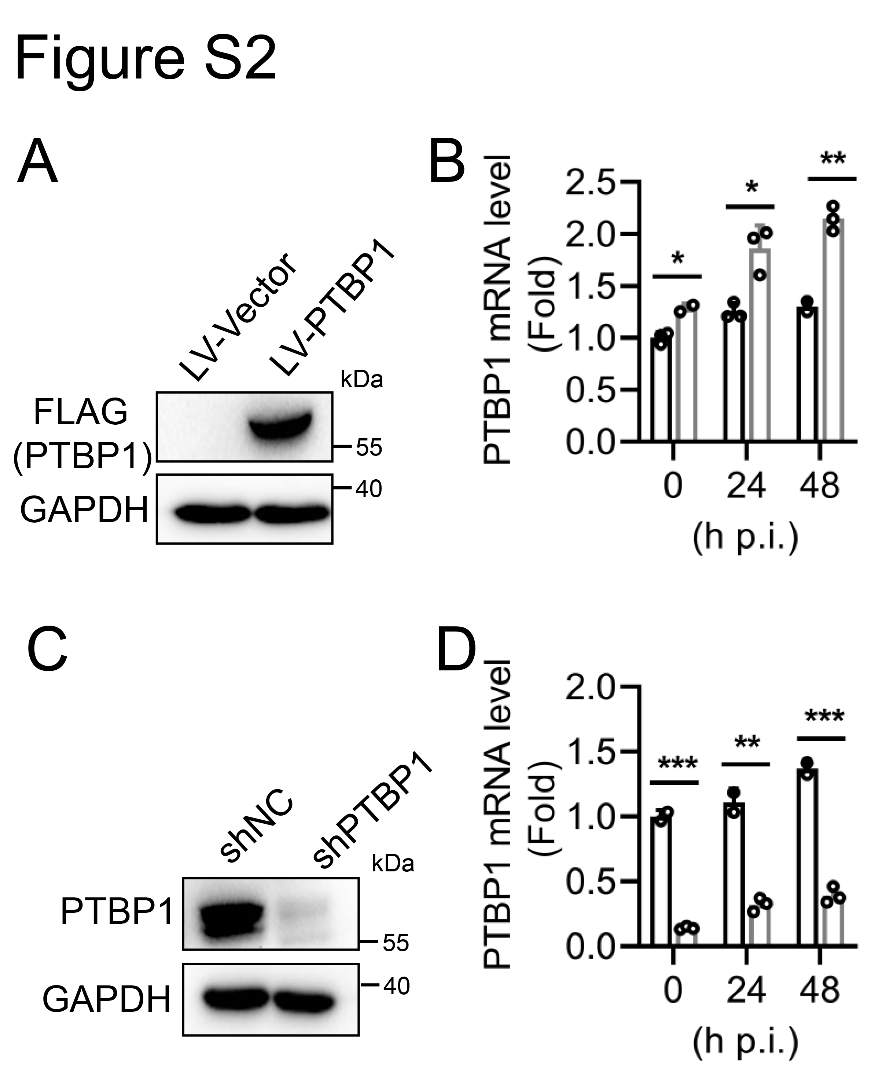


**Fig. S2 The expression of PTBP1 in PTBP1 -overexpressing and -knocking-down cells.**

(**A**) LV-PTBP1 and LV-Vector control cells were subjected to Western blot analysis to detect the expression of FLAG-tagged protein. (**B**) LV-PTBP1 and LV-Vector cells were infected with ZIKV (MOI = 1) at different time points (0, 24, and 48 h). Cells were collected at the corresponding time points for qRT-PCR analysis. (**C**) The stable expressing shPTBP1 and shNC control cells were subjected to Western blot analysis. (**D**) shPTBP1 and shNC cells were infected with ZIKV (MOI = 1) at different time points (0, 24, and 48 h). Cells were collected at the corresponding time points for qRT-PCR analysis. Graphs were expressed as Mean±SD, n = 3. ns, not-significant; *, P < 0.05; **, P < 0.01; ***, P < 0.001.


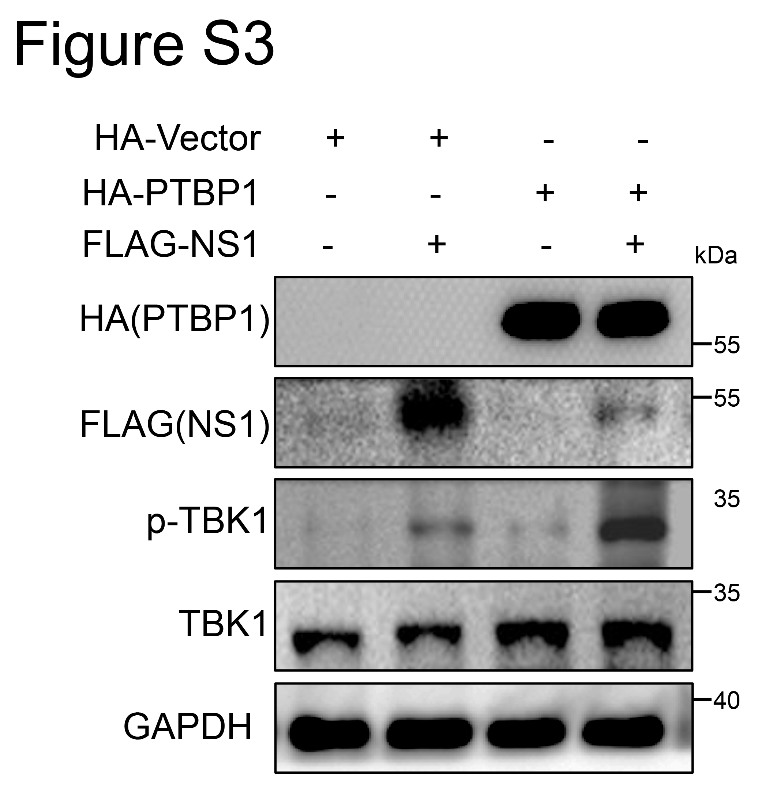


**Fig. S3 PTBP1-mediated ZIKV NS1 degradation enhances TBK1 activation.**

A549 cells were transfected with 1.0 μg of each plasmid: HA-Vector or HA-PTBP1 along with FLAG-Vector or FLAG-NS1. At 24 h post-transfection, the cells were subjected to Western blot analysis.
